# Comparative evaluation of the microbial diversity and metabolite profiles of Japanese-style and Cantonese-style soy sauce fermentation

**DOI:** 10.3389/fmicb.2022.976206

**Published:** 2022-08-08

**Authors:** Guiliang Tan, Yi Wang, Min Hu, Xueyan Li, Xiangli Li, Ziqiang Pan, Mei Li, Lin Li, Ziyi Zheng

**Affiliations:** ^1^School of Material Science and Food Engineering, University of Electronic Science and Technology of China, Zhongshan Institute, Zhongshan, China; ^2^School of Environmental and Safety Engineering, Changzhou University, Changzhou, China; ^3^School of Health Industry, Zhongshan Torch Polytechnic, Zhongshan, China

**Keywords:** soy sauce, metagenome, flavor metabolite, metabolic pathways, functional microbes

## Abstract

Microorganisms play essential roles in flavor formation during soy sauce fermentation. Different soy sauce fermentation types significantly affect flavor formation. However, comparisons of microbial communities and metabolites between different fermentation types have been little studied. Here, we investigated variation in microbial communities, metabolite profiles, and metabolic pathways during Japanese-type (JP) and Cantonese-type (CP) fermentation. Free amino acids and volatile compound profiles varied significantly between fermentation types, with JP samples containing higher contents of esters (39.84%; *p* < 0.05), alcohols (44.70%; *p* < 0.05) in the 120 d fermentation samples. Volatile compound profiles varied significantly between fermentation types, with JP samples containing higher contents of esters, alcohols, and free amino acids (*p* < 0.05). Metagenomic analysis indicated that both JP and CP communities were dominated by *Tetragenococcus*, *Staphylococcus*, *Weissella* (bacteria), and *Aspergillus* (fungi), but the two communities varied differently over time. *Tetragenococcus* drastically increased in abundance throughout the fermentation (from 0.02 to 59.2%) in JP fermentation, whereas *Tetragenococcus* (36.7%) and *Staphylococcus* (29.7%) dominated at 120 d of fermentation in CP fermentation. Metagenomic functional profiles revealed that the abundances of most genes involved with carbohydrate, amino acid, and lipid metabolism exhibited significant differences between fermentation types (*p* < 0.05) during the middle to late fermentation stages. Furthermore, predicted metabolic pathways for volatile substance biosynthesis differed between JP and CP fermentation, likely explaining the differences in flavor metabolite profiles. In addition, most of the genes associated with flavor generation were affiliated with *Tetragenococcus*, *Weissella*, *Staphylococcus*, *Bacillus*, and *Aspergillus*, suggesting that these microbes play important roles in flavor production during soy sauce fermentation. This study significantly improves our understanding of microbial functions and their metabolic roles in flavor formation during different soy sauce fermentation processes.

## Introduction

**Figure 1:**
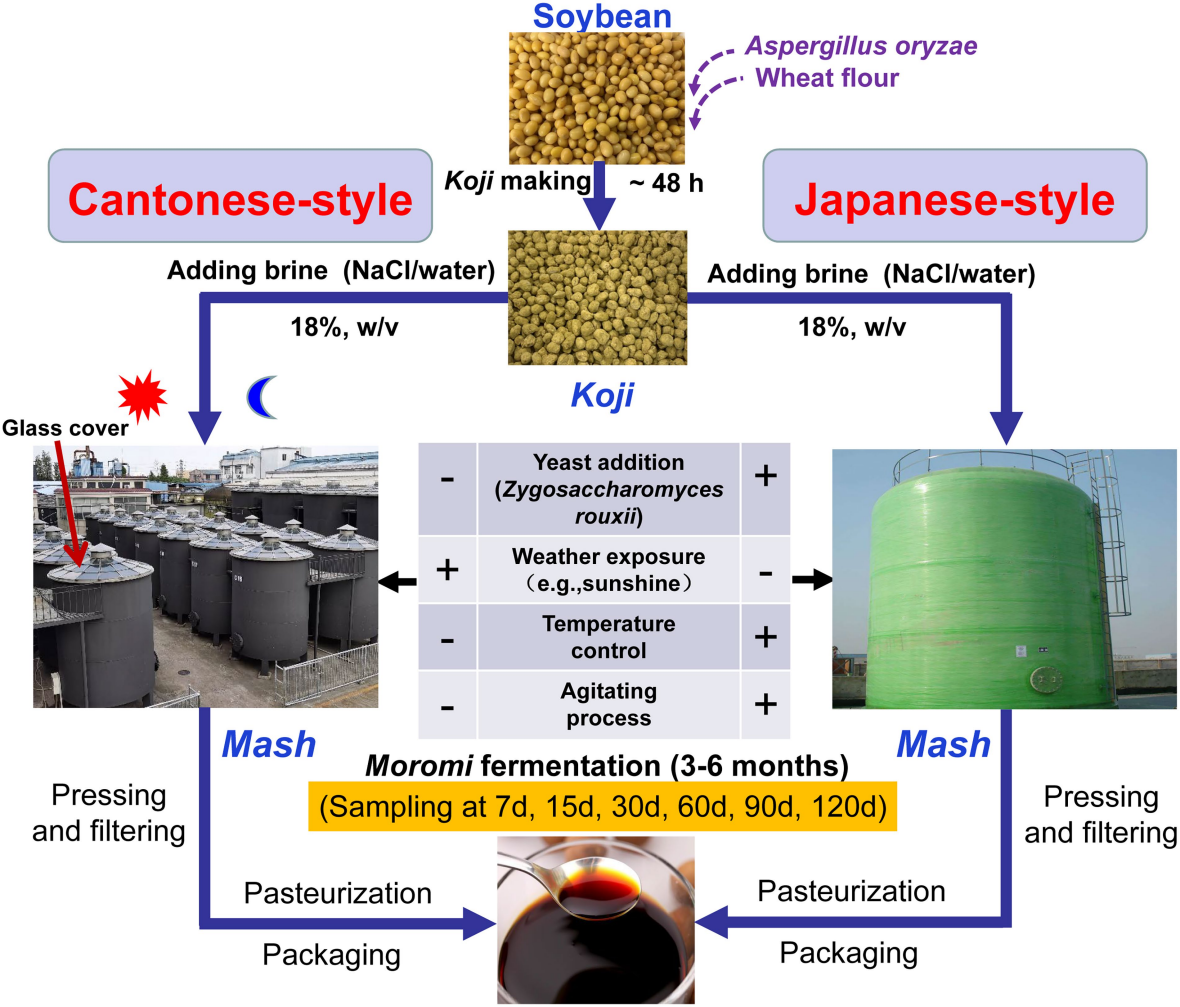
Schematic showing the process of soy sauce manufacturing in Japanese-type (JP) and Cantonese-type (CP) soy sauce production in addition to sampling points of this study.

Soy sauce is a traditional fermented soybean food product that originated in China and is the most widely consumed seasoning in China and other Asian countries (Feng et al., 2013). The annual production of soy sauce in China is about 10 million tons (Xu et al., 2021), and accounts for more than 50% of the world’s total production (Yan et al., 2013). Except for Korean soy sauce produced with only fermented soybeans (called *meju*; Kim et al., 2021), soy sauce can generally be classified into Chinese- and Japanese-types (JP) based on the amount of wheat used, the starter culture, and fermentation environments (Devanthi and Gkatzionis, 2019). The former is produced using mostly soybeans and less wheat (ratios of 80:20 or 70:30 soybean to wheat) under ambient temperatures and open environments, whereas the latter is primarily produced using an equal amount of soybeans and wheat, and with the addition of yeast (such as *Zygosaccharomyces rouxii* and *Candida* species) as starters under controlled temperature and aeration fermentation systems (Gao et al., 2019, 2020; Qi et al., 2021). JP soy sauce exhibits a high quality of flavor and is more widely consumed internationally due to higher amounts of alcohols, esters, and phenols (Feng et al., 2015). Cantonese-style (CP) soy sauce accounts for more than 70% of the Chinese-type soy sauce market and is more popular among Chinese consumers in China owing to its flavor (Gao et al., 2021; Liu et al., 2021). Although the two fermentation processes employ different strategies, they share a common two-step fermentation stage that includes *koji* generation and *moromi* fermentation (Devanthi and Gkatzionis, 2019; Figure 1).

Soy sauce fermentation is complex. During soy sauce fermentation, many flavoring substances are produced, including non-volatile compounds (e.g., free amino acids and organic acids) and volatile flavor compounds (VFCs; Diez-Simon et al., 2020). These flavoring substances are the most important factors that determine soy sauce quality and marketability (Wang et al., 2021). More than 300 VFCs have been identified in *moromi* mash, raw soy sauce, and commercial soy sauce (Feng et al., 2013; Meng et al., 2014). Among them, alcohols, esters, acids, and aldehydes are the most abundant aromatic compounds (Diez-Simon et al., 2020). The flavoring compound profiles of soy sauce vary depending on factors including the microbial strains, raw materials, salt concentrations, fermentation vessels, and fermentation temperatures used (Devanthi and Gkatzionis, 2019). Although the VFCs of CP and JP commercial soy sauce and soy sauce mash have been widely studied, few studies have compared the VFCs or other metabolites between CP and JP soy sauces across fermentation stages.

Microorganisms play essential roles in flavor production during soy sauce fermentation (Devanthi and Gkatzionis, 2019). Previous studies have shown that bacterial genera such as *Weissella*, *Staphylococcus*, *Tetragenococcus*, *Bacillus*, *Lactobacillus*, *Streptococcus*, *Enterococcus*, *Kurthia*, and *Klebsiella*, in addition to the fungal genera *Aspergillus*, *Zygosaccharomyces*, *Candida*, and *Debaryomyces*, primarily comprise soy sauce communities across the stages of fermentation (Sulaiman et al., 2014; Han et al., 2020; Qi et al., 2021). Metagenomics analyses have also revealed that microbial diversity decreases during the fermentation process, especially in the middle to late stages of *moromi* fermentation (Sulaiman et al., 2014; Chun et al., 2021; Kim et al., 2021). Furthermore, inferred functional profiles from metagenomic analyses have implicated the importance of the heterotrophic fermentation of proteins and carbohydrates during soy sauce production (Sulaiman et al., 2014). These metagenomics-based studies have enhanced our knowledge of the taxonomic diversity and functional potential of microbial populations involved in soy sauce fermentation. In addition, increased focus has been recently placed on associations between microbial community compositions and metabolites during soy sauce fermentation (Liu et al., 2021; Qi et al., 2021; Zhao et al., 2021). These studies have begun to elucidate the roles of microorganisms responsible for producing metabolites during specific soy sauce manufacturing processes. However, a comparative analysis of the microbial diversity, metabolite profiles, and metabolic pathways during different industrial soy sauce manufacturing processes has not yet been reported.

In order to understand the differences in volatilome profiles and the mechanisms responsible for these differences, the succession and metabolic functions of microbial communities as well as metabolite profiles during JP and CP industrial soy sauce production were investigated by coupling shotgun metagenomic and metabolomics approaches. In particular, the differences in the metabolic pathways and functional microbes associated with volatile substance biosynthesis were also specifically evaluated between JP and CP fermentation. To the best of our knowledge, this is the first report to comprehensively explore the differences of microbiomes and metabolome between different fermentation types. These results contribute to a better understanding of the effects of fermentation conditions on microbial succession and metabolite changes and the roles of microorganisms in flavor generation during different types of soy sauce fermentation, while helping to optimize soy sauce production to generate the highest quality soy sauce products.

## Materials and methods

### Sample preparation and sampling

Soy sauce production was conducted at a local factory (the Pearl River Bridge Biotechnology Co., Ltd.; Zhongshan, Guangdong, China) that is one of the most prominent seasoning manufacturing companies in China. The two manufacturing process types are illustrated in Figure 1. Before *koji* production, defatted soybeans were steam cooked and then mixed with wheat flour at a ratio of 3:1 (w/w), followed by cooling to 30°C and inoculation with 0.03% *Aspergillus oryzae* strain 3.042 as the spore starter. The mixtures were subsequently fermented to obtain *koji* in a *koji* manufacturing room at 28°C–35°C with 97–100% humidity. The *koji* was then immersed in a brine solution (approximately 18–22% NaCl concentration, w/v) to produce *moromis* in a 90 m^3^ fermenter at either natural temperature (CP; approximately 18°C to 22°C) or at a controlled temperature (JP; brine: *koji* = 2.5:1, w/w). The controlled temperature for JP fermentation was 15°C from 1 d to 30 d of fermentation, 30°C from 30 to 60 d, and 25°C from 60 to 120 d. *Z. rouxii* was used as the starter for JP fermentation after 30 d, and mashes were periodically agitated. After 4 months of fermentation, ripened *moromi* was subjected to a refining process that included pressing, filtering, pasteurization, and packaging to obtain commercial soy sauce (Figure 1). Mash samples from *moromi* fermentation were collected at days 7, 15, 30, 60, 90, and 120 during CP and JP fermentation. At each sampling time, mash samples were randomly collected from three different tanks as biological triplicates and placed in 50-ml centrifuge tubes (Corning CentriStar, NY, United States), immediately transported on ice to the laboratory, and then stored at −20°C before subsequent DNA extraction and chemical analysis.

### Chemical analyses of standardized form samples

Mash pH was measured with a PB-10 pH meter (Sartorius, Gottingen, Germany). The total acidity (TA), NaCl, and amino acid nitrogen (AAN) contents were analyzed using the titration method with an automatic potentiometric titrator (905-Titrando; Metrohm, Switzerland), as previously described (Tan et al., 2020). Free amino acid (FAA) contents were measured using ultra-high-performance liquid chromatography tandem MS (UPLC-MS/MS; model 1290/6460; Agilent Ltd., CA, United States), as previously described (Tan et al., 2020). After the samples were extracted with distilled water and the extracted solutions were purified using hexane (Merck, Darmstadt, Germany), the FAAs were separated on an ACQUITY UPLC BEH HILIC (2.1 × 100 mm, 1.7 μm; Waters Corp., MA, United States) using ammonium formate-acetonitrile/ammonium formate-H_2_O (pH 3.0) as the mobile phase. FAAs were then measured with MS/MS under multiple reaction monitoring modes.

The VFCs in mash samples were extracted using headspace solid-phase microextraction (SPME) and analyzed semi-quantitatively with gas chromatography–mass spectrometry (GC/MS), as previously described (Feng et al., 2013), but with minor modifications. Briefly, mash samples (2.5 g) were mixed with 0.5 g NaCl and 20 μl of an internal standard (2-methyl-3-heptanone at 2 mg/l in methanol) in 15 ml amber SPME vials, followed by equilibration with a thermostatic water bath at 55°C for 15 min. VFCs were then extracted with an SPME fiber (CAR/PDMS, 75 μm; Supelco Co., Bellefonte, PA, United States) at 55°C for 30 min. The VFCs were analyzed using a GC–MS system (model 6,890 N/5975; Agilent Ltd., CA, United States). The GC–MS oven temperature gradient started at 33°C (2 min), increased at 5°C/min to 70°C, then increased at 10°C/min to 250°C. The GC–MS settings included an injector temperature of 250°C and a run time of 30 min. Compounds were identified by comparing the mass spectral data against the NIST 14 mass spectral database. All extractions were conducted in triplicate. Semiquantitative data (μg/kg) were obtained by comparing their *m/z* peak areas to that of the internal standard on the GC–MS total ion chromatograms. All samples for chemical analysis mentioned above were analyzed in triplicate from three different tanks.

### DNA extraction and metagenomic sequencing

Total genomic DNA from mash samples (0.5 g) was extracted using an EZNA^™^ Mag-Bind Food DNA extraction kit (Omega Bio-Tek, Inc., Norcross, GA, United States) according to the manufacturer’s instructions. DNA quantity and quality were assessed with a Qubit 2.0 fluorometer (Invitrogen, Carlsbad, CA, United States) and with 1% agarose gel electrophoresis, respectively. Triplicate extracted DNA samples from the same sampling time were pooled and stored at −20°C for downstream metagenomic sequencing. DNA was sheared into approximately 350-bp fragments using a Covaris M220 nucleic acid shearer (Covaris, Woburn, MA, United States). Sequencing libraries were then constructed using a NEBNext Ultra DNA Library Prep Kit (NEB, Ipswich, MA, United States) according to the manufacturer’s protocols. The libraries were then sequenced on the NovaSeq 6,000 platform (Illumina Inc., San Diego, CA, United States) at the Novogene Bioinformatics Technology (Beijing, China), resulting in 2 × 150-bp paired-end sequencing reads.

### Bioinformatics analyses

Adapter sequences were removed from the generated reads, which were then trimmed using Trimmomatic v.0.30 with a quality cutoff of 30, a sliding window of 6 bp, and a minimum length cutoff of 45 bp (Bolger et al., 2014). High-quality reads were combined and assembled using IDBA-UD v.1.1.1 (Peng et al., 2012) to obtain contigs using the following parameters: minimum k value = 60; maximum *k* value = 120; increment of *k*-mer of each iteration = 10; minimum multiplicity for filtering *k*-mers when building the graph = 5; seed *k*-mer size for alignment = 5; minimum length of the contig = 1,000; and all other parameters were set to default values. Contigs shorter than 500 bp were excluded from further analyses. To utilize as many reads as possible during assembly, the unassembled reads were merged for a second assembly. Genes of the assembled contigs were predicted using the MetaGeneMark program (Zhu et al., 2010), with genes shorter than 300 nt removed from the dataset. CD-HIT software (Fu et al., 2012) was used to remove redundant genes at a 95% identity threshold and ≥ 90% coverage, with the longest sequence from each gene cluster used for downstream analysis as the representative sequence. Non-redundant genes were aligned to the National Center for Biotechnology Information (NCBI)-nr database using DIAMOND (Buchfink et al., 2015) with a threshold e-value ≤1 × 10^−5^, followed by taxonomic profiling using MEGAN (Huson et al., 2007). To determine the relative abundances of genes in each sample, the filtered reads were mapped back to the genes with Bowtie2 (Langmead and Salzberg, 2012) using default parameters. The read-mapping data were used to obtain the tags per million reads values for genes by calculating the abundances of the non-redundant gene profiles for each metagenome. The functional composition of the microbial communities was then obtained by comparing non-redundant gene sets against the Kyoto Encyclopedia of Genes and Genomes (KEGG; Kanehisa and Goto, 2000) database using the KOBAS v.3.0 program with an assignment threshold of e^−5^ (Xie et al., 2011). Metabolic pathways associated with dominant flavor compound formation were specifically evaluated with reconstructions based on the predicted KEGG pathways. Microbial populations that participated in the KEGG pathways were then identified based on taxonomic and functional annotations. When a gene associated with a specific microbial taxon was simultaneously annotated as an enzyme coding gene, an association between the enzyme and the microbial populations was inferred (Liu et al., 2019).

### Statistical analysis

All statistical analyses were conducted in the SPSS 18.0 software package (SPSS Inc., Chicago, IL, United States). Differences in data were tested by one-way analysis of variance (ANOVA) tests followed by a least significant difference test. Differences were considered statistically significant at a *p* < 0.05. Principal component analysis (PCA) was used with the *prcomp* function of R (Oksanen et al., 2013) to statistically compare the microbial community compositions and metabolite profiles for the two fermentation types. Linear discriminant analysis (LDA) effect size (LEfSe) was used to determine the most discriminatory taxa among the two different types of manufacturing processes using the LEfSe program v.1.0 (Segata et al., 2011). LEfSe was conducted with threshold values for the statistical test equal to 0.05 and a logarithmic LDA score threshold of 4.0.

### Sequence accessions

The sequence data for the metagenomes in this study are publicly available in the NCBI BioProject under accession no. PRJNA795848.

## Results and discussion

### Changes in chemical characteristics during *moromi* fermentation

Changes in chemical characteristics during *moromi* fermentation are shown in Supplementary Figure S1. The pH decreased from 7 to 60 d of fermentation, and then stabilized in later stages of JP and CP fermentation, ranging from 6.13 ± 0.06 to 4.82 ± 0.03 and 6.11 ± 0.10 to 4.81 ± 0.13, respectively. Salt contents remained relatively stable, ranging from 14.11 ± 0.96 to 13.88 ± 0.93% in JP fermentation and from 16.29 ± 0.78 to 15.15 ± 0.65 in CP fermentation. However, total acid (TA) and amino acid nitrogen (AAN) contents increased. The TA contents in JP and CP fermentation increased from 2.85 ± 0.16 to 3.83 ± 0.23 g/100 g (an increase of 1.3 times), and from 1.36 ± 0.16 to 3.48 ± 0.17 g/100 g (an increase of 2.6 times), respectively, whereas AAN contents increased from 1.26 ± 0.16 to 1.83 ± 0.13 g/100 g (a 1.5-fold increase) and 0.62 ± 0.06 to 1.70 ± 0.13 g/100 g (a 2.7-fold increase), respectively. AAN content is related to the proteolysis of proteins and has been considered a primary index to classify the quality of soy sauce products (Liu et al., 2021). The low contents of AAN in CP during the early fermentation period (7 to 15 d) compared to JP fermentation might reflect lower microbial activities responsible for proteolysis.

Differences in metabolites including free amino acids (FAAs) and VFCs were also evaluated. Among FAA profiles, amino acid contents significantly increased in the initial phase (from 7 to 15 d of fermentation), while most amino acids increased across the entire process (Supplementary Table S1). The arginine, lysine, and histidine contents drastically increased from 7 d to 30 d of fermentation, followed by decreases until the final stages (Supplementary Table S1). Among the 16 amino acids, phenylalanine (13.76 ± 0.32 g/kg in JP and 15.94 ± 0.25 g/kg in CP), leucine (JP: 16.88 ± 0.17 g/kg; CP: 20.44 ± 0.00 g/kg), and aspartic acid (JP: 21.17 ± 0.03 g/kg; CP: 16.04 ± 0.45 g/kg) were the predominant amino acid species at the end of fermentation (120 d). However, phenylalanine and leucine concentrations in CP (averages of 13.15 g/kg and 15.98 g/kg, respectively) were slightly higher than in JP fermentation (averages of 16.49 g/kg and 19.72 g/kg, respectively) during the middle to late stages of fermentation (from 60 to 120 d). Ten FAAs (leucine, tyrosine, proline, threonine, glycine, glutamic acid, aspartic acid, arginine, lysine, and histidine) exhibited significant differences in content between JP and CP samples at 120 d (*p* < 0.05; Supplementary Table S2). Among them, the concentrations of nine FAAs (except for leucine) in JP samples were significantly higher than those in CP samples. In particular, the umami (glutamic acid and aspartic acid) and sweet-tasting amino acids (proline, threonine, glycine, and lysine) were rich in JP samples at the end of fermentation (Supplementary Table S2).

**Figure 2:**
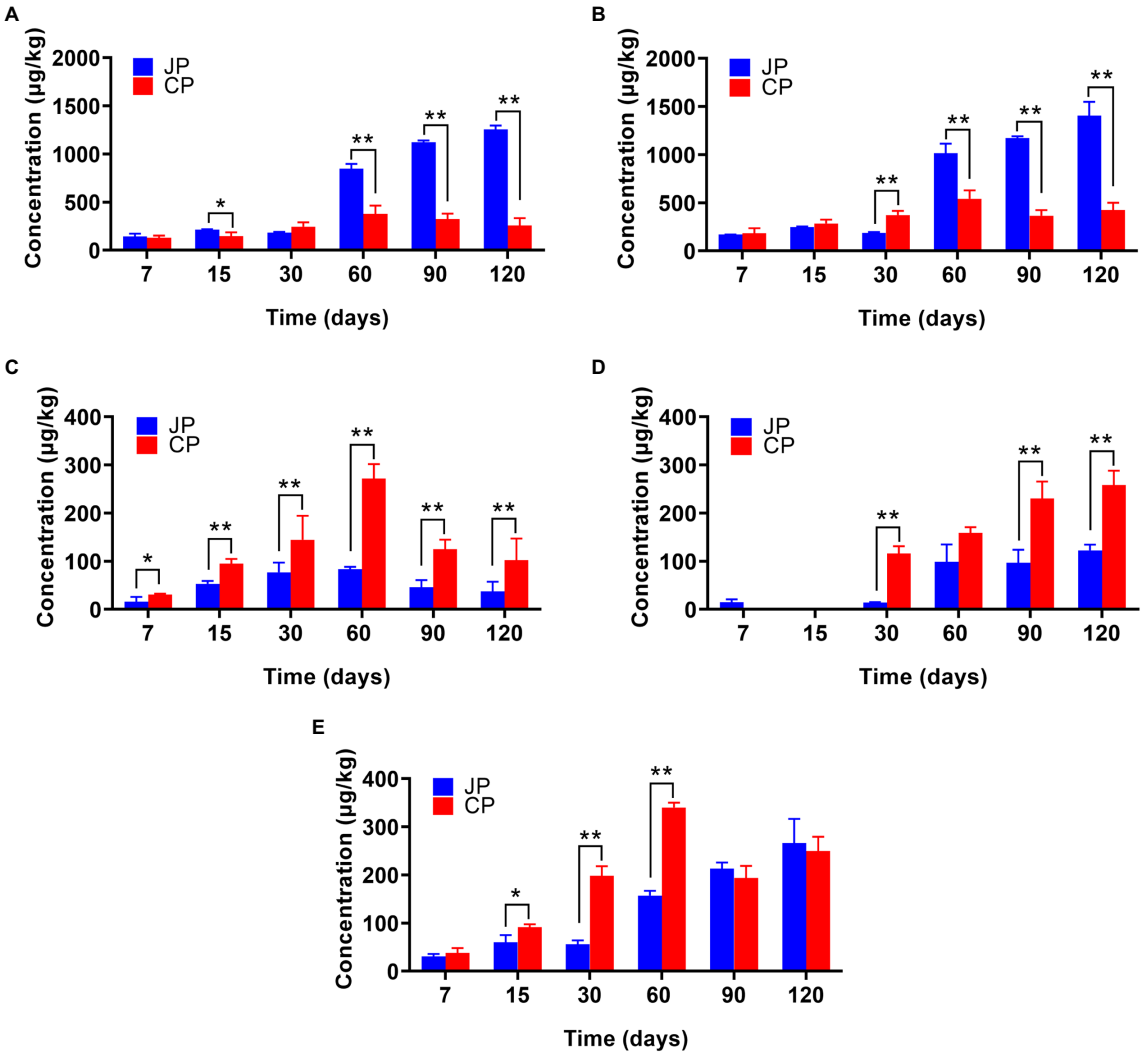
Abundances of various classes of volatiles in the two different soy sauce fermentation processes including esters **(A)**, alcohols **(B)**, phenols **(C)**, acids **(D)**, and aldehydes **(E)**. The averages ± SD of samples in each group are expressed in each column. ^*^p < 0.05; ^**^p < 0.01. JP, Japanese-type; CP, Cantonese-type.

A total of 85 VFCs were identified during *moromi* fermentation, including 32 esters, 16 alcohols, 10 aldehydes, two acids, four phenols, and 21 other compounds (i.e., alkanes, ketones, furan(one)s, and pyrazines; Supplementary Table S3). The contents of these VFC types gradually increased during fermentation and remained steady at later stages (Figure 2). However, the levels of the dominant VFC types significantly differed between fermentation, with higher contents of esters and alcohols in JP compared to CP in the middle to late fermentation stages (*p* < 0.05), while higher contents of phenols and acids were observed in CP fermentation (*p* < 0.05; Figure 2). Higher amounts of esters and alcohols contribute significantly to the flavor of Japanese-type soy sauce (Feng et al., 2015). In the 120 d fermentation samples, esters (an average of 39.84% of total VFC concentrations in JP and 17.94% in CP), alcohols (JP: 44.70%; CP: 29.60%), phenols (JP: 1.19%; CP: 7.14%), acids (JP: 3.89%; CP: 18.02%), and aldehydes (JP: 8.48%; CP: 17.43%) were the predominant volatile groups identified (Supplementary Table S3). However, the identified volatiles species were significantly different, especially for esters and alcohols. The most abundant VFCs in the JP samples were ethyl acetate (an average of 218.78 μg/kg in the later three stages), ethyl caproate (134.84 μg/kg), ethanol (373.42 μg/kg), 1-butanol (153.41 μg/kg), and phenylethyl alcohol (215.66 μg/kg). In contrast, the major VFCs in the CP samples included ethyl acetate (138.70 μg/kg), 1-octen-3-ol (290.33 μg/kg), acetic acid (209.16 μg/kg), 4-ethylguaiacol (62.13 μg/kg), and 4-vinylguaiacol (82.76 μg/kg; Supplementary Table S3). A total of 28 high-abundance VFCs (out of the 30 detected) were significantly different between soy sauce types at 120 d of fermentation, including ethyl acetate, ethyl caproate, ethanol, 1-butanol, 3-methyl-1-butanol, 1-octen-3-ol, phenylethyl alcohol, acetic acid, and 4-vinylguaiacol (*p* < 0.05; Supplementary Table S2). Many esters (e.g., ethyl acetate, ethyl caproate, ethyl benzoate, ethyl caprylate, ethyl phenylacetate, and ethyl palmitate) and alcohols (e.g., ethanol, 1-butanol, 3-methyl-1-butanol, and phenylethyl alcohol) were identified at relatively high levels in JP mash samples compared with CP samples during later fermentation stages. Phenylethyl alcohol, which is floral and sweet-tasting, has been reported to be one of the main compounds linked to the floral notes of soy sauce (Diez-Simon et al., 2020). Esters are responsible for fruity notes (Diez-Simon et al., 2020) and predominantly accumulate during intermediate stages of fermentation (e.g., at 60 d), becoming more abundant in the last fermentation phase (Figure 2A). The formation of esters in JP might be partially related to the lipid metabolism of yeast (*Z. rouxii*) or other micoorganisms (e.g., LAB) that provide abundant acids and alcohols, which may subsequently generate a variety of esters through microbial esterification (Lee et al., 2006; Feng et al., 2015). In addition, these differences in the contents and species of VFCs between the two fermentation processes may be due to the different proportions of wheat used and the environmental conditions during fermentation, such as temperature and oxygen (Diez-Simon et al., 2020), as well as the different microbial communities present in the fermentation systems. For example, lower contents of ethanol in CP can be ascribed to the lack of a major carbohydrate source because only small amounts of are wheat used (Diez-Simon et al., 2020). The principal component analysis (PCA) of the metabolites measured, including the amino acids and VFCs, further revealed a large distinction in metabolite profiles between the two fermentation types (Supplementary Figure S2A). The metabolite profiles of JP and CP mash were similar before 30 d of fermentation, but were clearly distinguished after 30 d of fermentation, suggesting that different microbial communities might have contributed to the generation of different flavor compound profiles. Notably, two metabolite profile samples (at 90 and 120 d of fermentation) clustered together for JP and CP fermentation, suggesting that most flavor compounds were produced after 90 d of fermentation.

In this study, we found volatile compound profiles varied significantly between fermentation types, with JP samples containing higher contents of esters, alcohols (Figure 2), and FAAs (Supplementary Table S2; *p* < 0.05). The divergency of the volatile compound profiles between CP and JP fermentation must have resulted from these two manufacturing process. Besides temperature control, agitation should be another process that influences the production of the volatile compound during soy sauce fermentation. It is recognized that agitation is a critical parameter and plays a significant role in determining the productivity of vinegar (Hutchinson et al., 2019). During soy sauce fermentation, agitation can improve the mash mixing and heat transfer in the tank. In addition, agitation also improves the oxygen transfer rate in the liquid system, benefiting to increase the microbial growth and activity in fermentation.

### Dynamics of microbial communities across soy sauce fermentation types

To identify the composition and succession of microbial communities during fermentation within the two different soy sauce manufacturing processes, metagenomic sequencing was conducted on 12 mash samples (six stages for each manufacturing process). A total of 64.5 Gbp of sequencing data was retained after quality filtering, with an average of 5.38 Gbp for each sample (ranging from 5.0 to 5.7 Gb; Table 1). A large fraction of sequence reads was assembled into contigs ≥500 bp, yielding an N50 (defined as the minimum contig length needed to cover 50% of the total contig length) from 2,138 to 18,035 per sample (with a maximum contig length of 760,846 bp and a mean contig size of 2,031 bp). Thus, assemblies were successfully produced from the quality-filtered reads, with average alignment rates of 85.27% ± 2.16% for the 12 samples (Table 1). The assembled contigs therefore possessed most of the genetic information of the soy sauce mash microbial communities. The abundances of bacterial-associated sequences increased from 59.3 to 98.5% across the JP fermentation stages, whereas fungus-affiliated sequences concomitantly decreased from 34.3 to 0.70%. In addition, the abundance of bacteria increased from 77.2 to 96.3% across the CP fermentation stages, whereas fungus-affiliated sequences concomitantly decreased from 18.2 to 0.70% (Table 1). No archaeal-affiliated sequences were observed in the metagenomic data.

**Table 1:** Sequencing, assembly information, and taxonomic composition of soy sauce mash samples.

Sample	No. of sequences	Total sequence length (Gbp)	%GC	Alignment rate (%)	No. of contigs	Total contig length (bp)	Max. contig length (bp)	Avg. contig length (bp)	N50	No. of ORFs	Bacteria (%)	Fungi (%)	Unclassified (%)
JP7d	18,335,141	5.3	45	87.44	30,999	88,696,625	694,737	2,861	18,035	95,583	59.29	34.33	6.38
JP15d	18,930,296	5.6	44	85.65	49,454	119,998,478	390,056	2,426	8,163	140,817	91.53	5.31	3.16
JP30d	18,240,344	5.3	44	87.44	42,854	109,808,458	760,838	2,562	9,912	126,397	77.27	18.30	4.43
JP60d	17,119,640	5.0	42	81.81	44,948	87,370,354	406,621	1,944	3,378	114,172	96.97	1.10	1.94
JP90d	19,057,073	5.6	41	86.77	37,819	70,539,343	290,526	1,865	3,117	95,671	98.53	0.17	1.30
JP120d	19,047,838	5.5	42	86.72	40,865	71,170,104	545,028	1,742	2,862	96,188	97.48	0.71	1.81
CP7d	19,435,323	5.7	49	81.76	109,112	218,415,514	621,643	2,002	4,511	282,485	77.22	18.19	4.60
CP15d	18,583,624	5.5	48	86.94	70,112	164,691,919	760,846	2,349	6,230	201,962	85.04	11.49	3.47
CP30d	17,407,000	5.0	48	85.24	73,968	165,177,457	361,554	2,233	5,518	206,962	90.74	5.38	3.89
CP60d	17,362,384	5.1	45	86.12	71,800	119,541,980	404,088	1,665	2,370	154,302	95.26	1.97	2.77
CP90d	18,572,263	5.5	46	81.98	84,238	128,677,542	328,607	1,528	2,138	178,908	96.14	1.30	2.56
CP120d	18,490,542	5.4	43	85.37	51,963	91,343,735	269,489	1,758	3,793	122,050	96.26	0.74	3.01

**Figure 3:**
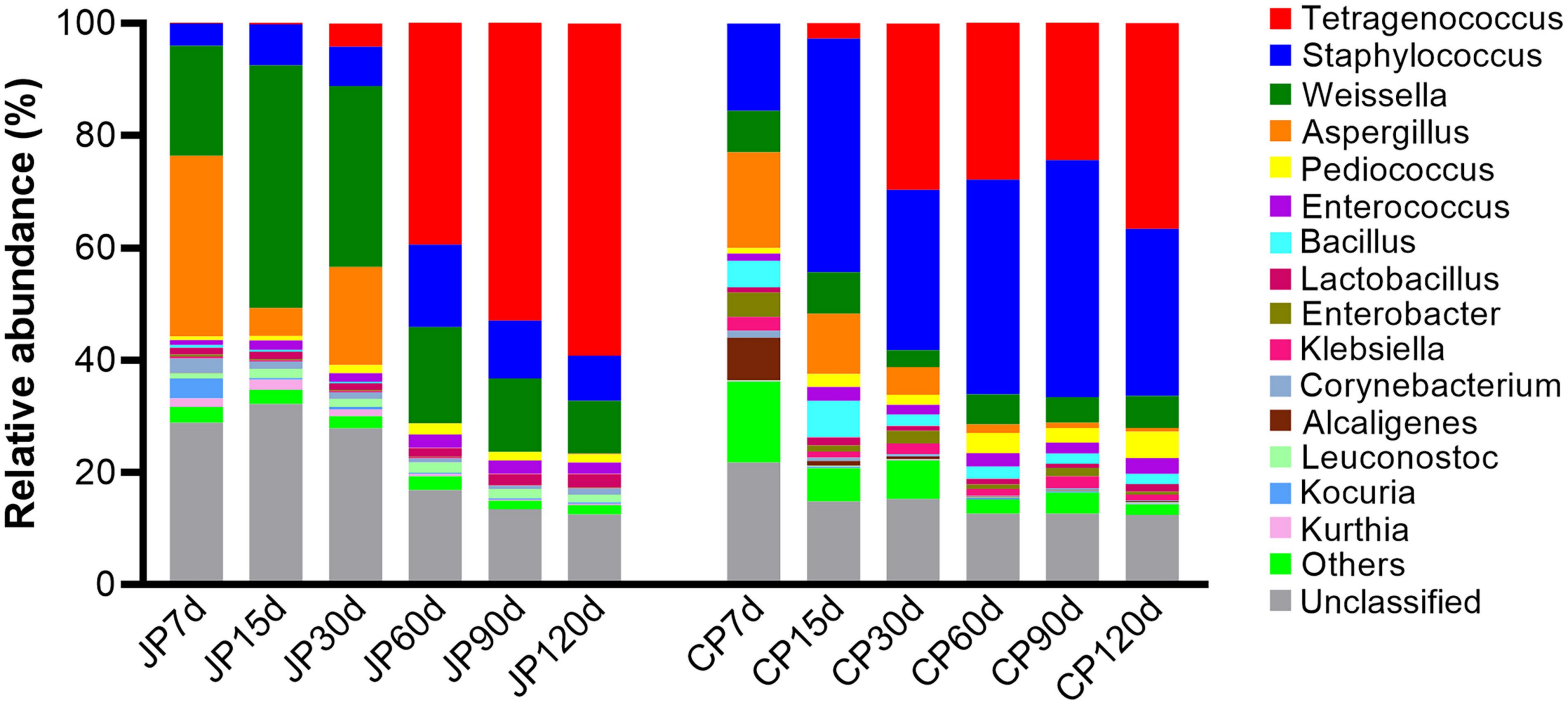
Microbial taxonomic composition at the genus level for the 15 most abundant genera across the two different fermentation processes. Taxonomic profiles were obtained using the MEGAN software package by comparison against the NCBI-nr database. “Others” comprise the less-abundant genera. Sequences that could not be matched to any known taxonomic groups are designated as “unclassified.” Samples are labeled according to fermentation time (7, 15, 30, 60, 90, and 120 d) and type (JP, Japanese-style fermentation; CP, Cantonese-style fermentation).

Sequences affiliated with the families *Enterococcaceae*, *Staphylococcaceae*, *Leuconostocaceae*, *Aspergillaceae*, *Lactobacillaceae*, *Enterobacteriaceae*, and *Bacillaceae* were dominant during both JP and CP fermentation (Supplementary Figure S3). However, the relative abundances of the fungal family Aspergillaceae dramatically decreased with fermentation (from 33.0 to 0.13% in JP and from 17.5 to 0.67% in CP). At the genus level, the bacterial genera *Tetragenococcus*, *Staphylococcus*, and *Weissella*, in addition to the fungal genus *Aspergillus*, dominated JP communities. *Tetragenococcus* drastically increased in abundance throughout the fermentation (from 0.02 to 59.2% of all sequences), whereas *Aspergillus* populations declined, with abundances of 32.1% at 7 d of fermentation to 0.12% at 120 d of fermentation (Figure 3). In CP fermentation, the genera *Tetragenococcus* and *Staphylococcus* dominated at 120 d of fermentation, representing 36.7 and 29.7% of the sequences at 120 d, respectively, followed by *Weissella* (5.75%) and *Pediococcus* (4.65%). The relative abundance of *Aspergillus* was also very low at the end of CP fermentation (0.65%), similar to that observed in JP fermentation. The relative abundances of *Bacillus* were higher in CP communities (average 3.16%) than in JP communities (0.22%). Notably, *Tetragenococcus* dominated the middle to late stage JP fermentation communities, whereas *Tetragenococcus* and *Staphylococcus* were dominant in the middle to late stages, with *Staphylococcus*, in particular, dominating all CP fermentation stages (Figure 3). The high relative abundances of *Tetragenococcus* in JP samples (60–120 d of fermentation) compared to those in CP samples might be related to the higher temperature (25°C), which is favorable for the growth of these microbes. In contrast, the lower temperature (18°C–22°C at 60–120 d of fermentation) in CP might suppress the growth of *Tetragenococcus*. This finding was in accordance with previous reports in which the fermentation temperature was found to be an important factor influencing the growth of *Tetragenococcus* during saeu-jeot fermentation (Lee et al., 2014). The higher relative abundances of *Staphylococcus* in CP might be related to higher salt contents (Supplementary Figure S1), in accordance with previous results (Han et al., 2020).

*Tetragenococcus*, a halophilic lactic acid bacteria (LAB), has been identified as the dominant microbial taxa in *moromi*, and is involved in the production of lactate, acetate, and ethanol (Chun et al., 2021). The particularly high abundances of *Tetragenococcus* in both the JP and CP samples suggest that *Tetragenococcus* may play an essential role in flavor development during soy sauce fermentation. Most *Staphylococcus* species are harmless to humans and are widely used in traditional sauce products to enhance volatile flavor compound production (Guo et al., 2020). *Weissella* species are common obligate LAB that have been detected in a variety of fermented foods and play important roles in flavor generation (e.g., by producing lactic acid, isoamyl acetate, and terpinyl acetate; Xiang et al., 2020). The high abundances of these species might originate from *koji* materials, because they are dominant in finished *koji* communities after 48 h of *koji* production. *Bacillus* spp. are also dominant bacterial taxa in many fermented soybean products (Devanthi and Gkatzionis, 2019; He and Chung, 2020) and contribute to flavor generation during soy sauce aging through amylase and protease activities (Wei et al., 2013; Liang et al., 2019). *Bacillus* spp. can also improve the nutritional values of soy products (Shi et al., 2017). The higher abundances of *Bacillus* present in CP compared to those in JP was likely related to more anaerobic conditions in CP fermentation due to the lack of agitation during fermentation, suggesting that *Bacillus* species prefer anaerobic or microaerophilic conditions. A previous study reported that *Bacillus* species (such as *B. subtilis*) may grow anaerobically (Nakano and Zuber, 1998). *Aspergillus* species play important roles in flavor formation during soy sauce fermentation, particularly in *koji* generation and the early stages of *moromi* fermentation (Tanaka et al., 2012; Devanthi and Gkatzionis, 2019). The relative abundances of *Aspergillus* were high in the first three stages owing to the use of the *A. oryzae* starter (an average of 18.2% in JP and 10.93% in CP), and later became minor populations (lower than 0.65% in the final stage; Figure 3). This observation was similar to those from Ganjang (soy sauce) samples, where *Aspergillus* abundances were very low (Chun et al., 2021), suggesting that *Aspergillus* is not an important taxon during the middle to late stages of soy sauce fermentation. These decreases are mainly due to the high salinity content of *moromi*, which is unfavorable for *Aspergillus* growth because *Aspergillus* species are not halotolerant (Sassi et al., 2021). Other fungal yeasts such as *Zygosaccharomyces*, *Candida*, *Pichia*, *Kluyveromyces*, *Saccharomyces*, and *Wickerhamiella* were detected at very low abundances throughout both types of fermentation.

In Japanese style soy sauce mash, *Z. rouxii* appeared in the early fermentation stage (Tanaka et al., 2012; Xu et al., 2021) since it was inoculated as starer at this stage. It is reasonable in this study that the *Zygosaccharomyces* was not detected at 30 days of fermentation, because it was just added as a starter and propagated slowly at this stage. However, it is unexpected that *Zygosaccharomyces* was detected with low abundance at 60 days of fermentation, which is inconsistent with previous study, where the relative abundance of *Zygosaccharomyces* (added at 30 days) increased rapidly at 60 days, reach the highest abundance, then decreased dramatically after 90 days (Xu et al., 2021). The low abundance of *Zygosaccharomyces* in the present study might be closely related to the sampling time point. The high abundance of *Zygosaccharomyces* might appear within 45 days of fermentation, then decreased or disappeared after this stage. Therefore, a more reasonable sampling time point is indispensable for the detection of *Zygosaccharomyces*.

To further explore differences in microbial community structures between JP and CP fermentation, biomarker analysis was performed with the linear discriminant analysis (LDA) effect size (LEfSe) method and an LDA threshold score of 4.0, which have been previously suggested as suitable for the statistical analysis of metagenomic data from two or more microbial communities (Segata et al., 2011). LEfSe revealed that *Tetragenococcus, Weissella* (genus), Lactobacillales (order), and *Leuconostocaceae* (family) were abundant in JP fermentation samples, whereas Bacillales (order), *Staphylococcus* (genus), *Staphylococcaceae* (family), and *Pediococcus* (genus) were especially abundant in CP communities (Supplementary Figure S4). PCA analyses based on genus-level compositional differences were performed to evaluate differences in communities among mash samples. Samples from JP and CP fermentation clustered separately (Supplementary Figure S2B), suggesting significant differences in the taxonomic profiles of JP and CP fermentation communities. The apparent differences in microbial community compositions from different fermentation processes can be primarily explained by environmental factors such as the temperature, photoperiod, and oxygen availability during fermentation, which differed between JP and CP fermentation processes, because the same *koji* was used for both types of fermentation.

The relationships between the microorganisms and metabolites during two soy sauce fermentation processes were determined (Supplementary Figure S5). The results showed that the abundances of *Tetragenococcus* and *Pediococcus* were positively correlated with FAAs (all *p* < 0.05). *Tetragenococcus* was also positively correlated with most of VFCs (all *p* < 0.05), suggesting that this bacterium may play a vital role in soy sauce fermentation. Three high-abundant substances (e.g., ethanol, ethyl acetate, and phenylethyl alcohol) were all positively correlated with *Tetragenococcus* (*p* < 0.01). The abundance of 1-octen-3-ol was positively correlated with *Pediococcus*, *Enterococcus*, *Staphylococcus*, *Ochrobactrum*, *Bacillus*, and *Klebsiella* (*p* < 0.05). Besides, positive correlations between 4-ethyl guaiacol (4-EG) with *Pediococcus*, *Staphylococcus*, and *Tetragenococcus*, 4-vinylguaiacol (4-VG) with *Pediococcus*, *Enterococcus*, and *Staphylococcus*, acetic acid with *Pediococcus* and *Tetragenococcus* were observed during whole soy sauce fermentation, respectively (*p* < 0.05). *Tetragenococcus* was also found to be correlated with the production of 1-octen-3-ol, 4-EG, glutamic acid, acetate, and ethanol in *moromi* (Chun et al., 2021; Qi et al., 2021). Collectively, our study together with another research (Qi et al., 2021), both unveiled the important role of T*etragenococcus*, *Pediococcus* and *Staphylococcus* in the production of 4-EG. Unexpectedly, the abundances of *Aspergillus* were only positively related to methyl palmitate, but negatively related to most metabolites (*p* < 0.05; Supplementary Figure S5). In addition, the abundance of *Weissella* was only positively correlated with two low-content level metabolites [tyrosine and (R,R)-2,3-butanediol; *p* < 0.05].

### Changes in microbial functional potential

**Figure 4:**
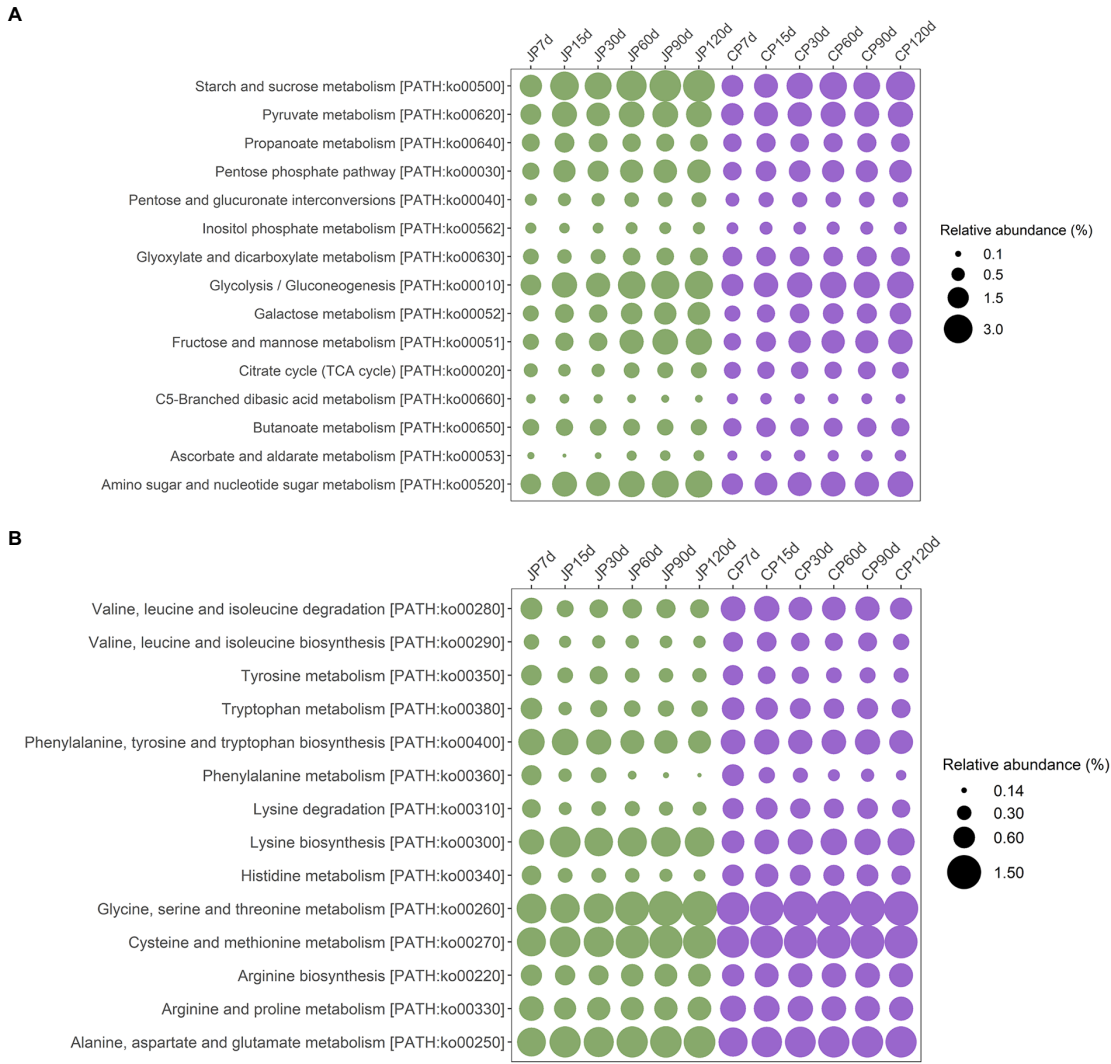
Variation in the functional gene categories associated with carbohydrate metabolism **(A)** and amino acid metabolism **(B)** from soy sauce mash microbiota based on metagenomic annotation for the JP and CP fermentation types. Functional classes were determined according to the level 3 KEGG annotations using whole shotgun metagenome-derived ORFs. JP, Japanese-type; CP, Cantonese-type; KEGG, Kyoto Encyclopedia of Genes and Genomes; ORFs, open reading frames.

The metabolic characteristics of the two types of soy sauce microbiomes were investigated using Kyoto Encyclopedia of Genes and Genomes (KEGG) classifications. Based on the KEGG level 2 pathways, genes associated with metabolism were the most abundant (Supplementary Figure S6). Within the metabolism category, the most abundant metabolic type was carbohydrate metabolism (an average of 16.78% in JP communities and 16.84% in CP communities), followed by amino acid metabolism (10.01% in JP communities and 10.76% in CP communities). The higher abundances of genes associated with carbohydrate metabolism and amino acid metabolism suggest that starch and protein substrates serve as critical flavor precursors. However, amino acid metabolism gene abundances differed between the two fermentation communities, with dramatically decreased abundances in JP communities from 7 d of fermentation onward, and then becoming stable in later fermentation stages, while their abundances in CP metagenomes decreased more slowly (Supplementary Figure S6). At level 3 of KEGG classification, several subsets of carbohydrate metabolism genes were enriched in both JP and CP communities that were involved in starch and sucrose metabolism (ko00500), pyruvate metabolism (ko00620), glycolysis/gluconeogenesis (ko00010), fructose/mannose metabolism (ko00051), and amino sugar and nucleotide sugar metabolism (ko00520) as fermentation proceeded (Figure 4A). Among the aforementioned groups, starch and sucrose metabolism (ko00500), pyruvate metabolism (ko00620), glycolysis/gluconeogenesis (ko00010), and amino sugar and nucleotide sugar metabolism (ko00520) genes were in higher abundance in the JP communities (averages of 3.63, 2.35, 2.82, and 2.57%, respectively) than in the CP communities (2.79, 2.23, 2.50, and 2.18%, respectively) during the middle to late fermentation stages (i.e., at 60 to 120 d). Differential abundance analysis revealed that the abundances of all genes associated with carbohydrate metabolism at KEGG level 3 (except for those involved in fructose and mannose metabolism [ko00051]) were significantly different between JP and CP communities (*p* < 0.05; Supplementary Table S4). The high-abundance genes related to carbohydrate metabolism in JP samples might derive from the high abundances of *Tetragenococcus* and *Weissella* in JP samples. A previous study also observed that carbohydrate metabolism related-genes from *Tetragenococcus* were highly expressed, with *Tetragenococcus* inferred to be mostly responsible for carbohydrate metabolism during Ganjang (soy sauce) fermentation (Chun et al., 2021).

The dominant genes involved in amino acid metabolism were involved in lysine biosynthesis (ko00300); glycine, serine, and threonine metabolism (ko00260); cysteine and methionine metabolism (ko00270); and alanine, aspartate, and glutamate metabolism (ko00250; Figure 4B). Among these, only genes involved in lysine biosynthesis (ko00300) exhibited significantly different abundances between the JP and CP metagenomes (*p* < 0.05) in the middle to late fermentation stages (Supplementary Table S4). The abundances of these genes remained stable, suggesting that mash microbiomes encoded a high potential for metabolizing these amino acids. Interestingly, other low-abundance genes that encoded proteins involved in valine, leucine, and isoleucine biosynthesis (ko00290), tyrosine metabolism (ko00350), tryptophan metabolism (ko00380), phenylalanine metabolism (ko00360), lysine degradation (ko00310), and histidine metabolism (ko00340) exhibited decreased abundances across JP and CP fermentation (Figure 4B). In particular, the abundances of these genes greatly decreased at 15 d of fermentation for both JP and CP communities (Figure 4B). The abundances of these genes also differed significantly between the JP and CP metagenomes (*p* < 0.05) in the middle to late stages of fermentation. The different amino acid metabolism profiles of JP and CP microbiomes might result in different concentrations and profiles of amino acids, such as those for leucine, tyrosine, arginine, lysine, and histidine, which exhibited significant differences between JP and CP fermentation (*p* < 0.05; Supplementary Table S1). Overall, amino acid metabolism was more prevalent in CP fermentation communities than in JP fermentation communities (Figure 4B; Supplementary Figure S5), which may have resulted from the higher abundances of *Staphylococcus* in CP communities (Figure 3), consistent with previous observations (Hu et al., 2021).

Differences in lipid metabolism were also evaluated between the two types of soy sauce manufacturing processes. Genes associated with glycerophospholipid metabolism (ko00564; an average of 0.81% overall, but accounting for 20.89% of lipid metabolism genes), glycerolipid metabolism (ko00561; 0.98 and 25.17%), and fatty acid biosynthesis (ko00061; 1.03 and 26.35%) were most abundant (Supplementary Figure S7). Lipid metabolism may influence the final *kokumi* characteristics of soy sauce owing to the myriad metabolite byproducts that come from lipid degradation (Diez-Simon et al., 2020). However, these high-abundance genes did not significantly differ in abundances (*p* ≥ 0.05) between the JP and CP communities in the middle to late stages of fermentation (Supplementary Table S4). In contrast, some low-abundance genes involved in the synthesis and degradation of ketone bodies (ko00072) and the biosynthesis of unsaturated fatty acids (ko01040) exhibited significant differences between soy sauce fermentation types (*p* < 0.05).

### Differences in metabolic pathways between the two manufacturing processes

**Figure 5:**
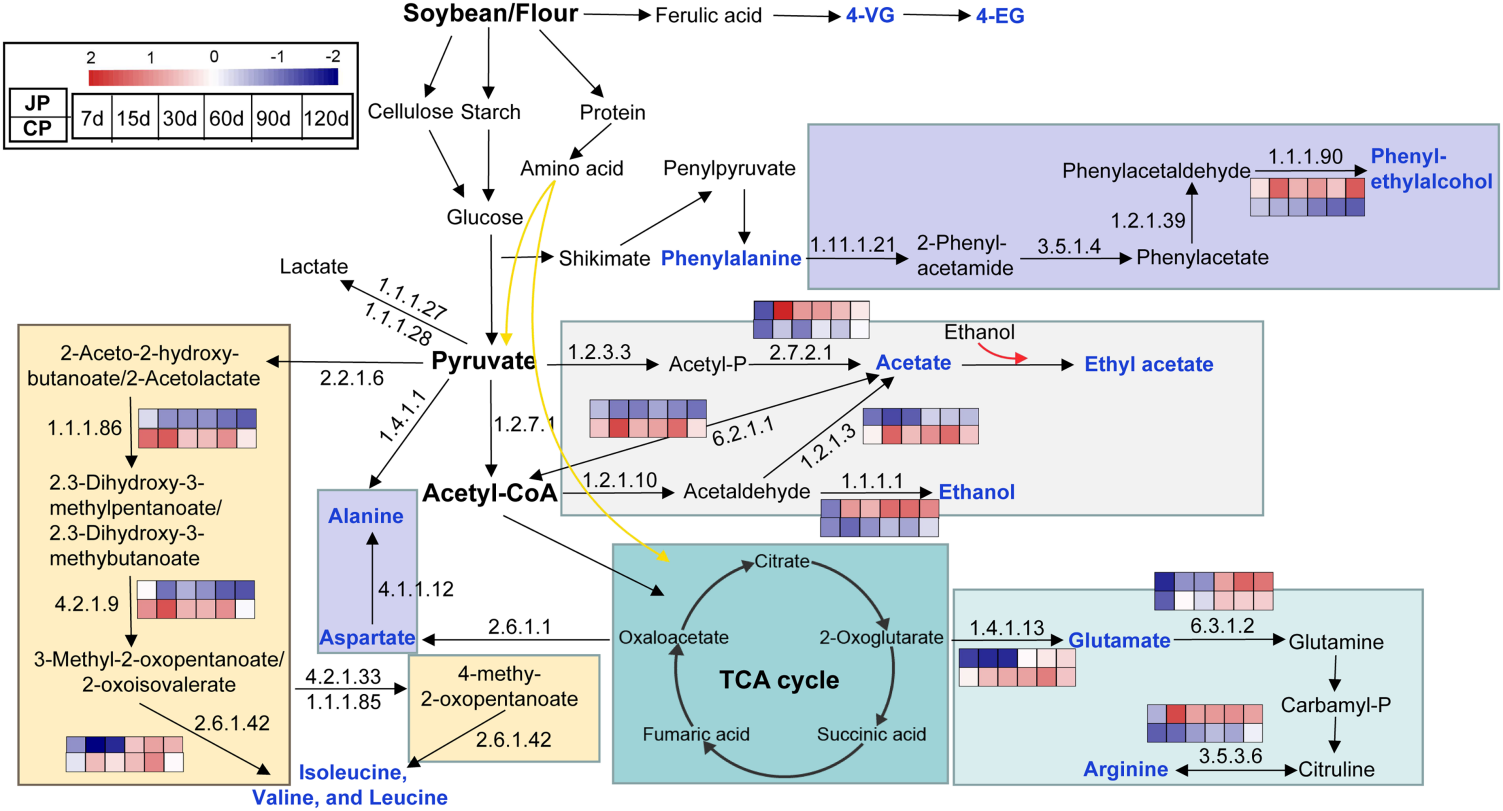
Predicted metabolic pathways involved in the formation of dominant flavors during soy sauce fermentation. Flavor metabolites are labeled in blue. The abundance changes of KEGG genes are shown across different fermentation times. Heatmap values range from +2 to −2, indicating high to low abundance, respectively. JP, Japanese-type; CP, Cantonese-type; KEGG, Kyoto Encyclopedia of Genes and Genomes.

The metagenomic and metabolomics data (Supplementary Tables S1, S3, S5) were used to further predict and reconstruct pathways associated with the metabolism of flavoring compounds (Figure 5). The profiles of genes encoding enzymes involved in the biosynthesis of flavoring substances differed between JP and CP fermentation. The abundances of genes encoding alcohol dehydrogenases (EC 1.1.1.1), aryl-alcohol dehydrogenases (EC 1.1.1.90), and arginine deiminases (EC 3.5.3.6) were higher in JP communities than in CP communities and were responsible for the production of ethanol, phenylethyl alcohol, and arginine, respectively. In contrast, genes encoding aldehyde dehydrogenases (EC 1.2.1.3) and acetyl-CoA synthetases (EC 6.2.1.1) involved in acetate biosynthesis were highly enriched in CP communities (Figure 5; Supplementary Table S5), corresponding with the higher acetate contents observed in CP fermentation (Supplementary Table S3). The amino acids in soy sauces may originate from proteins or peptides in the raw materials, or are otherwise synthesized by microorganisms (Devanthi and Gkatzionis, 2019). Genes encoding branched-chain amino acid aminotransferases (EC 2.6.1.42) exhibited higher abundances in CP communities than in JP communities between 7 to 90 d of fermentation. These differences might be attributed to the different microbial compositions observed between JP and CP fermentation. Specifically, the differences in the metabolic pathways could be correlated with the differences in the abundances of *Tetragenococcus, Weissella, Bacillus*, *Staphylococcus*, and *Pediococcus* (Supplementary Figure S4). The biosynthesis of esters lacks a resolved metabolic pathway in the KEGG database, but the production of higher ester contents such as ethyl acetate in the JP fermentation could be interpreted as being due to higher concentrations of ester precursors (alcohols and acids; Figures 2B,D). Interestingly, the biosynthesis of glutamate could be primarily contributed from glutamate synthase (NADPH) large-chain (EC 1.4.1.13) proteins rather than glutaminase (EC: 3.5.1.2; Supplementary Table S5), suggesting the presence of an unusual biosynthetic pathway of glutamate (Ito and Matsuyama, 2021). In addition, 4-VG and 4-EG were detected in both JP and CP mashes and were likely generated by the non-enzymatic degradation of ferulic acid (Mo and Xu, 2010; Liu et al., 2019).

### Relationships between microorganisms and enzymes in different metabolic pathways

**Figure 6:**
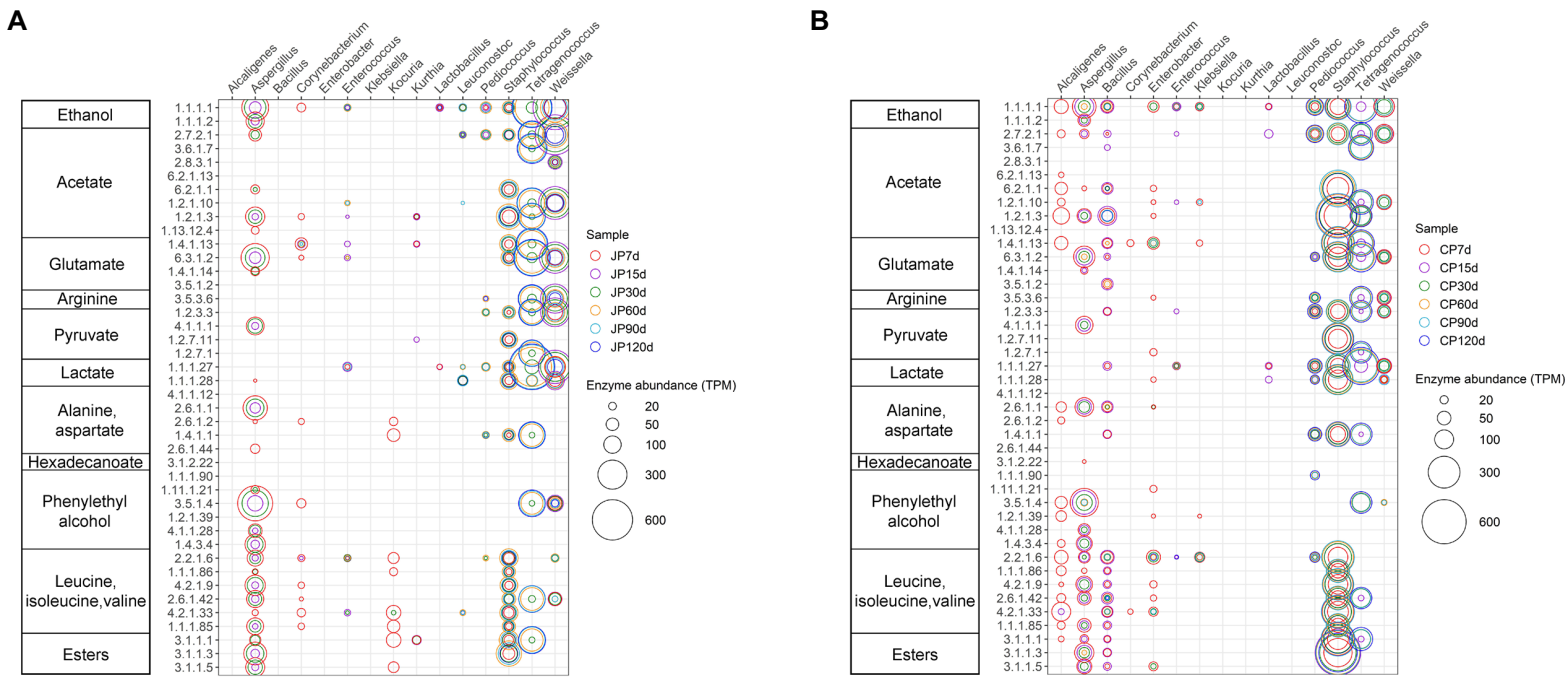
Taxonomic distribution and abundances of enzymes involved in substrate breakdown and flavor formation during soy sauce fermentation for JP **(A)** and CP **(B)** fermentation. Only enzymes from the 15 most abundant taxa were predicted. Circle diameter correlates to enzyme read numbers. Samples are labeled according to fermentation time (7, 15, 30, 60, 90, and 120 d) and type (JP, Japanese-style processes; CP, Cantonese-style processes).

To explore the differences in metabolic pathways between the JP and CP communities, relationships were identified among the enzymes and microorganisms involved in important metabolic pathways (Figure 6). The flavor formation and substrate breakdown of soy sauce mash were grouped into 11 functional assemblies. In particular, the abundances of enzymes encoded by six genera (*Tetragenococcus*, *Weissella*, *Staphylococcus*, *Pediococcus*, *Bacillus*, and *Aspergillus*) were higher; these enzymes were likely the most closely related to flavor production. However, the contribution of these microorganisms to the aforementioned flavoring substances differed between the JP and CP communities. *Tetragenococcus* and *Weissella* were the main contributors to flavoring compounds in JP fermentation, whereas *Staphylococcus* and *Tetragenococcus* were the main contributors for CP fermentation (Figure 6). *Tetragenococcus* might participate in the formation of certain flavoring substances, such as ethanol, acetate, arginine, glutamate, lactate, branched-chain amino acids (BCAAs, isoleucine, leucine, and valine), and esters. In addition, *Staphylococcus* species are the main microorganisms involved in generating acetate, glutamate, glutamine, lactate, BCAAs, and esters. *Tetragenococcus,* together with *Weissella,* might be involved in the heterolactic fermentation of proteins and carbohydrates during these two types of fermentation. This was suggested by the finding that they harbored high-abundance L-lactate dehydrogenases (EC:1.1.1.27), acetate kinases (EC:2.7.2.1), aldehyde dehydrogenases (NAD+; EC:1.2.1.3), and alcohol dehydrogenases (EC:1.1.1.1) that were associated with the production of lactate, acetate, and ethanol, respectively. The facultative heterolactic pathway of *Tetragenococcus* has also been implicated as a contributor to Ganjang (soy sauce) fermentation (Chun et al., 2021). In addition, LAB species are generally considered the primary producers of lactic acid. *Tetragenococcus* was the most important contributor to genes encoding L-lactate dehydrogenases (EC:1.1.1.27) in JP and CP (Figure 6), which suggested that *Tetragenococcus* may be mainly responsible for lactate production during soy sauce fermentation. However, genes involved in lactate synthesis were mapped not only to LAB *Pediococcus*, *Tetragenococcus*, and *Weissella* genomes, but also to non-LAB *Staphylococcus* and *Bacillus* genomes, which was consistent with a previous study of broad bean paste fermentation (Jia et al., 2021). Furthermore, *Staphylococcus* was the most important contributor to genes encoding the aldehyde dehydrogenases (NAD^+^; EC:1.2.1.3) responsible for acetate production during fermentation (especially in the CP fermentation; Figure 6). In addition, *Staphylococcus* was highly associated with BCAA production, harboring more genes involved in BCAA metabolic pathways (Figure 6). BCAAs are important for proteolysis in some LAB species and are essential in the production of volatile compounds such as acids, alcohols, and esters (Sulaiman et al., 2014; Chun et al., 2021). The high contribution of *Staphylococcus* to functional gene profiles was due to its high abundance within the fermentation communities (Figure 3) and its ability to use diverse substrates as sources of carbon, energy, and nitrogen (Jia et al., 2021).

*Bacillus* species were primarily abundant in CP fermentation and encoded various enzymes related to the production of ethanol, acetate, glutamate, and BCAAs. It is noteworthy that branched-chain amino acid aminotransferases (EC 2.6.1.42) were primarily associated with *Tetragenococcus*, *Staphylococcus*, and *Aspergillus* in the JP and CP communities. These enzymes were also associated with *Weissella* in the JP communities, but not *Weissella* in the CP communities, although high abundances of *Weissella* were observed across CP fermentation stages (an average of approximately 5.56%; Figure 3). This discrepancy could be due to the presence of different dominant *Weissella* species or strain-dependent differences among *Weissella* species that reflect different functional traits best adapted to these two different manufacturing process environments. *Aspergillus* might contribute to the formation of ethanol, acetate, glutamate, aspartate, phenylethyl alcohol, BCAAs, and esters during early fermentation periods, which also corresponds to the decreasing abundances of *Aspergillus* in our samples (Figure 3). In addition, annotation results showed that *Aspergillus*, *Bacillus*, and *Staphylococcus* had genes encoding esterases (carboxylesterase, EC 3.1.1.1) and lipases (triacylglycerol lipase, EC 3.1.1.3), while *Tetragenococcus* only had genes encoding carboxylesterase (EC 3.1.1.1). This finding suggests that these microbes may play a significant role in ester production by releasing fatty acids from triglycerides or synthesizing fatty acid esters (Holland et al., 2005).

## Conclusion

In this study, the differences in community composition and metabolic functions of microbiomes along with metabolite variation were systematically compared within and between two different types of soy sauce fermentation processes (JP and CP fermentation). The two fermentation processes harbored significantly different microbial communities (especially in the proportion of microbes, such as *Tetragenococcus*, *Staphylococcus*, and *Bacillus*) and flavor metabolites. Although mash samples from JP were characterized by higher contents of ester, alcohols, and FAAs (especially umami- and sweet-flavored amino acids) compared to those from CP, most flavor compounds (FAAs and VFCs) within JP and CP were largely produced after 90 d of fermentation, and their contents remained relatively stable. Distinct gene profiles associated with metabolic pathways were also apparent when comparing the JP and CP communities. Genes related to the metabolic pathways of starch and sucrose, glycolysis/gluconeogenesis, amino sugars, and nucleotide sugars were more abundant in JP communities than in CP communities during the middle to later stages of fermentation. Furthermore, the predicted metabolic pathways associated with the biosynthesis of volatile substances revealed different gene abundances in JP and CP fermentation, shaping the formation of some volatile substances. The relationships between enzymes and microorganisms within particular metabolic pathways revealed that there was an unusual biosynthetic pathway for glutamate production, and *Staphylococcus*, *Tetragenococcus*, and *Aspergillus* might contribute to the formation of esters. Fermentation temperature and oxygen conditions might be important factors influencing microbial growth. The present study provides comprehensive insights into the microorganisms involved in the generation of flavoring compounds during different soy sauce manufacturing processes. To better understand the functional roles of different species during *in situ* flavor production, future studies should evaluate gene expression and metagenome-assembled genomes with metatranscriptomics and genome-resolved metagenomics approaches. Further research on the effects of temperature on microbial succession and metabolite changes in different fermentation conditions (e.g., high temperatures, such as 35°C in summer) in CP fermentation will be indispensable and will help to better elucidate its potential importance.

## Data availability statement

The datasets presented in this study can be found in online repositories. The names of the repository/repositories and accession number(s) can be found at: https://www.ncbi.nlm.nih.gov/, PRJNA795848.

## Author contributions

GT and MH conceived and designed the experiments in addition to writing the manuscript. GT and YW conducted the experiments and data analyses. XuL and XiL performed most of the experiments. ZP, ML, LL, and ZZ supervised the execution of the experiments. All authors contributed to the article and approved the submitted version.

## Funding

This work was supported by the Natural Science Foundation of Guangdong Province (grant nos. 2020A1515011308, 2020A1515011577, and 2022A1515012158), the National Science Foundation of China (grant no. 41977138), the Construction Project of Teaching Quality and Teaching Reform in Guangdong Province (grant no. SJD202001), the General University Project of Guangdong Provincial Department of Education (grant nos. 2021KCXTD070 and 2021ZDZX4072), the Key Project of Social Welfare and Basic Research of Zhongshan City (grant no. 2020B2010), and the start-up fund from the Zhongshan Institute at the University of Electronic Science and Technology in China (grant no. 419YKQN12).

## Conflict of interest

The authors declare that the research was conducted in the absence of any commercial or financial relationships that could be construed as potential conflict of interest.

## Publisher’s note

All claims expressed in this article are solely those of the authors and do not necessarily represent those of their affiliated organizations, or those of the publisher, the editors and the reviewers. Any product that may be evaluated in this article, or claim that may be made by its manufacturer, is not guaranteed or endorsed by the publisher.

## Supplementary material

SUPPLEMENTARY FIGURE S1 Changes in physicochemical properties during soy sauce fermentation including pH **(A)**, NaCl concentrations **(B)**, TA contents **(C)**, and AAN contents **(D)**. Data are presented as means ± standard errors from triplicate measurements. JP, Japanese-type; CP, Cantonese-type; TA, total acidity; AAN, amino acid nitrogen.

SUPPLEMENTARY FIGURE S2 Principal component analysis (PCA) score plots for the composition of major metabolites produced **(A)** during JP and CP fermentation in addition to microbial community compositional variation at the genus level **(B)**. Metabolites in the analysis included all FAAs and the 30 most abundant VFCs that were detected. The microbial communities comprised the 20 most abundant genera. The percent variation of each principal component is indicated on their respective axes. The curved arrows indicate directions of variation change in metabolite and microbial communities for each fermentation process. JP, Japanese-type; CP, Cantonese-type; FAAs, free amino acids; VFCs, volatile flavor compounds.

SUPPLEMENTARY FIGURE S3 Taxonomic abundance profiles of microbial communities at the family level during different fermentation processes. Only the 10 most abundant families and genera are shown for ease of visualization. “Others” comprise the less-abundant families. Sequences that could not be assigned to a known taxonomic group are designated as “unclassified.” Samples are labeled according to fermentation time (7, 15, 30, 60, 90 and 120 d) and fermentation type (JP, Japanese-style process; CP, Cantonese-style process).

SUPPLEMENTARY FIGURE S4 Linear discriminant analysis (LDA) effect size (LEfSe) of microbial communities from different fermentation processes (JP, Japanese-type; CP, Cantonese-type). Differences in microbial community structures were analyzed using the LDA effect size algorithm. LDA scores represent the level of differentiation for a given group using a threshold score of 4.0.

SUPPLEMENTARY FIGURE S5 Heatmap of correlations among microbial genera and metabolites. Metabolites included all FAAs and the 30 most abundant VFCs that were detected. The microorganisms comprised the 20 most abundant genera. Correlation strength (based on Spearman’s *r*-value) and correlation significance values are shown as shaded colors (red, positive correlation; blue, negative correlation). Heatmap values range from + 1.0 to −1.0. Values above/below zero represent positive/negative correlations, respectively, between genera and parameters. ^*^*p* < 0.05, ^**^*p* < 0.01.

SUPPLEMENTARY FIGURE S6 Variation in the abundances of functional categories associated with carbohydrate metabolism **(A)** and amino acid metabolism **(B)** in soy sauce mash microbial communities based on metagenome annotations for two different fermentation types. Functional classes were determined at the level 2 classifications of KEGG annotations using whole shotgun metagenome assembly-derived ORFs. JP, Japanese-type; CP, Cantonese-type; KEGG, Kyoto Encyclopedia of Genes and Genomes; ORFs, open reading frames.

SUPPLEMENTARY FIGURE S7 Variation in the abundances of functional categories associated with lipid metabolism from soy sauce mash microbial communities based on metagenome annotations for two different fermentation processes. Functional classes were determined at the level 3 classifications of KEGG annotations using whole shotgun metagenome assembly-derived ORFs. JP, Japanese-type; CP, Cantonese-type; KEGG, Kyoto Encyclopedia of Genes and Genomes; ORFs, open reading frames.
