# Supplementary figures and images for: Comparative evaluation of the microbial diversity and metabolite profiles of Japanese-style and Cantonese-style soy sauce fermentation
Source: Front Microbiol. 2022 Aug 8;13:976206. doi: 10.3389/fmicb.2022.976206 (PMC9393507; doi:10.3389/fmicb.2022.976206)

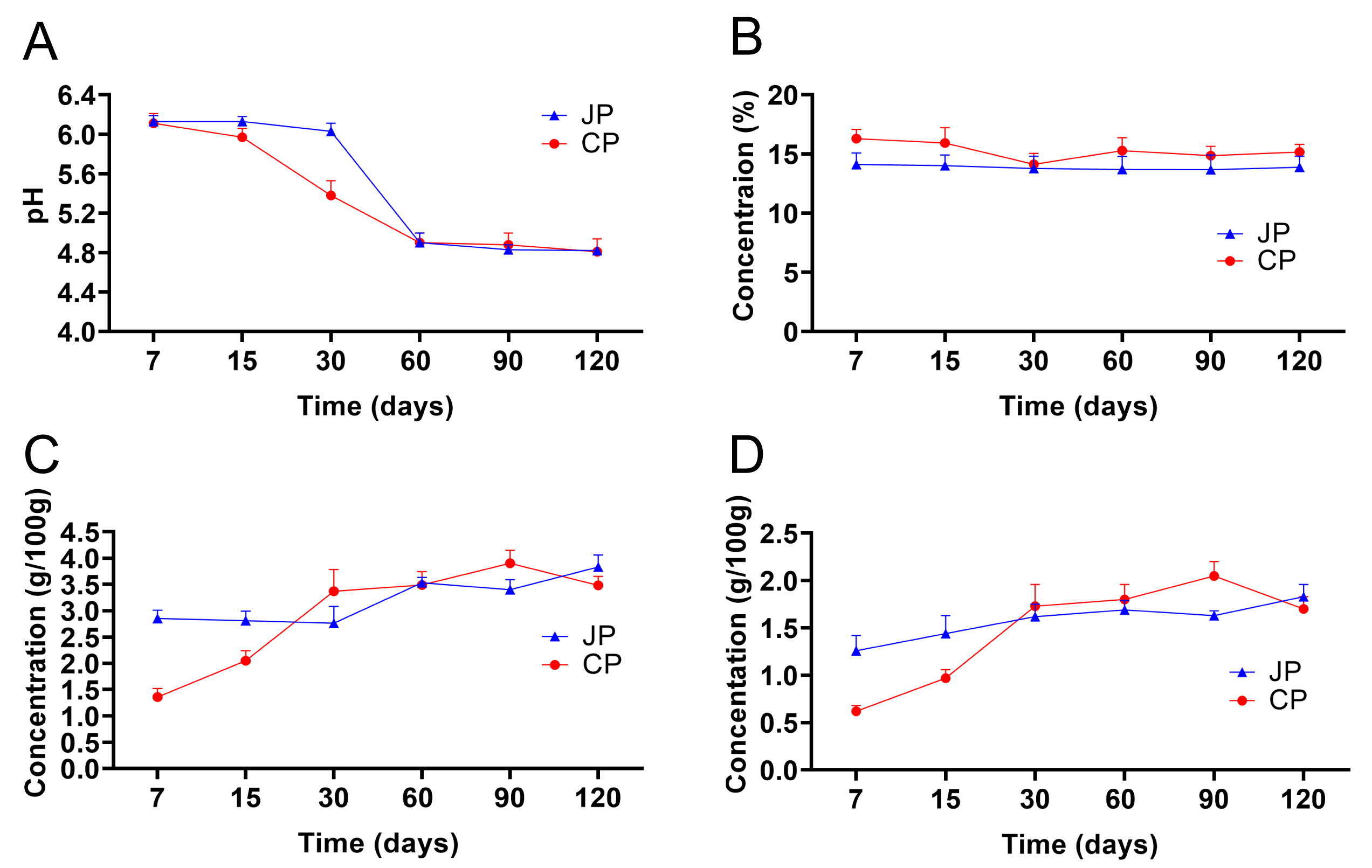

Supplement: SUPPLEMENTARY FIGURE S1 — Changes in physicochemical properties during soy sauce fermentation including pH (A), NaCl concentrations (B), TA contents (C), and AAN contents (D). Data are presented as means ± standard errors from triplicate measurements. JP, Japanese-type; CP, Cantonese-type; TA, total acidity; AAN, amino acid nitrogen. [file Image_1.TIF]

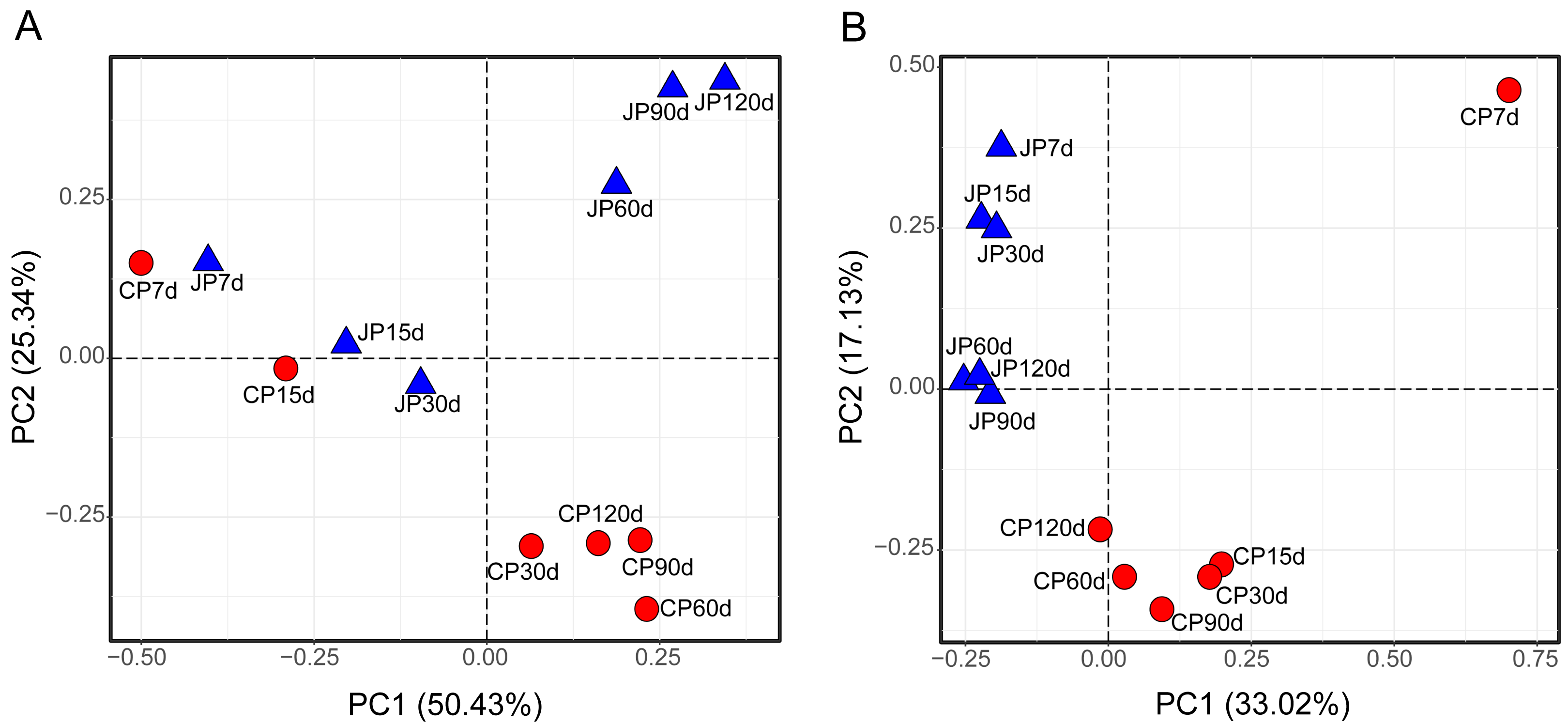

Supplement: SUPPLEMENTARY FIGURE S2 — Principal component analysis (PCA) score plots for the composition of major metabolites produced (A) during JP and CP fermentation in addition to microbial community compositional variation at the genus level (B). Metabolites in the analysis included all FAAs and the 30 most abundant VFCs that were detected. The microbial communities comprised the 20 most abundant genera. The percent variation of each principal component is indicated on their respective axes. The curved arrows indicate directions of variation change in metabolite and microbial communities for each fermentation process. JP, Japanese-type; CP, Cantonese-type; FAAs, free amino acids; VFCs, volatile flavor compounds. [file Image_2.TIF]

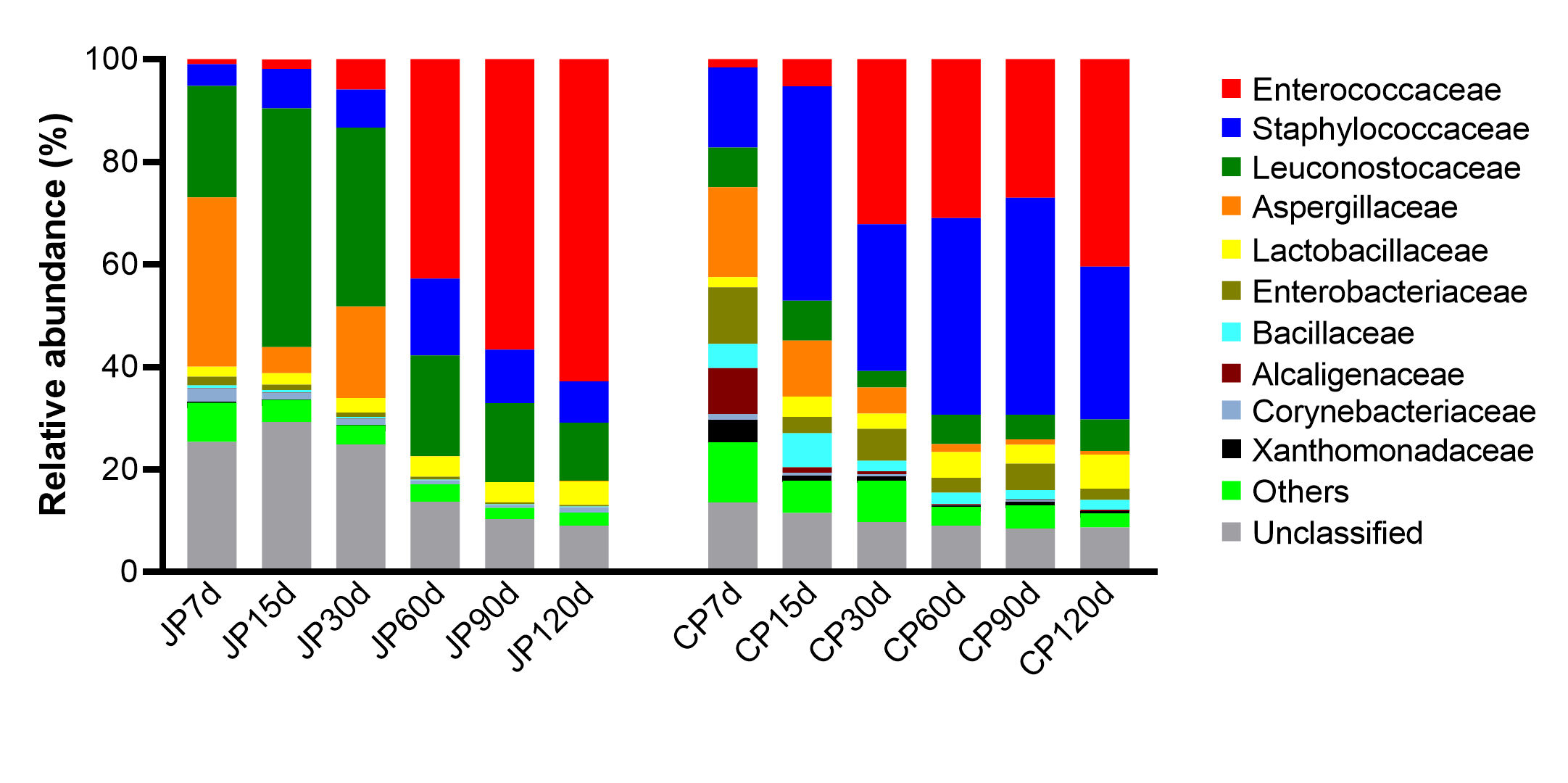

Supplement: SUPPLEMENTARY FIGURE S3 — Taxonomic abundance profiles of microbial communities at the family level during different fermentation processes. Only the 10 most abundant families and genera are shown for ease of visualization. “Others” comprise the less-abundant families. Sequences that could not be assigned to a known taxonomic group are designated as “unclassified.” Samples are labeled according to fermentation time (7, 15, 30, 60, 90 and 120 d) and fermentation type (JP, Japanese-style process; CP, Cantonese-style process). [file Image_3.TIF]

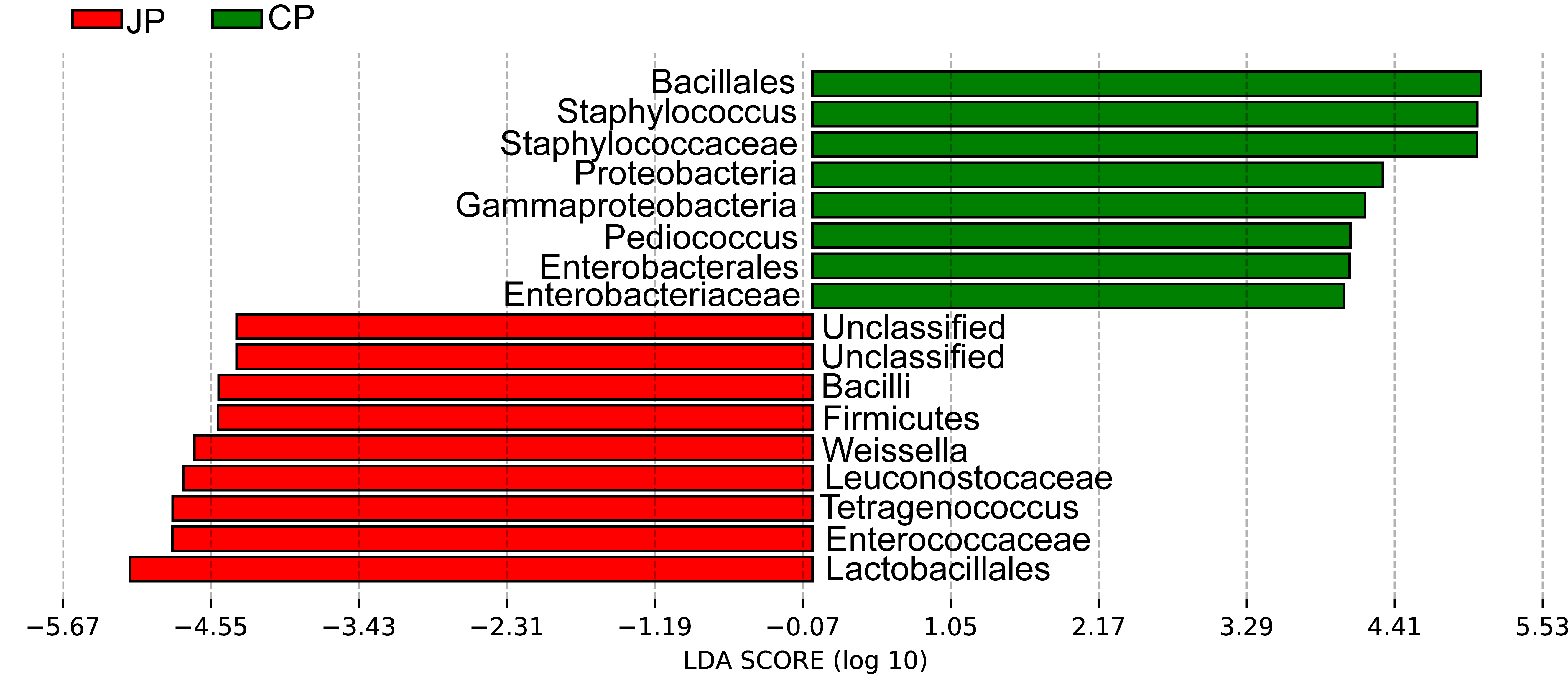

Supplement: SUPPLEMENTARY FIGURE S4 — Linear discriminant analysis (LDA) effect size (LEfSe) of microbial communities from different fermentation processes (JP, Japanese-type; CP, Cantonese-type). Differences in microbial community structures were analyzed using the LDA effect size algorithm. LDA scores represent the level of differentiation for a given group using a threshold score of 4.0. [file Image_4.TIF]

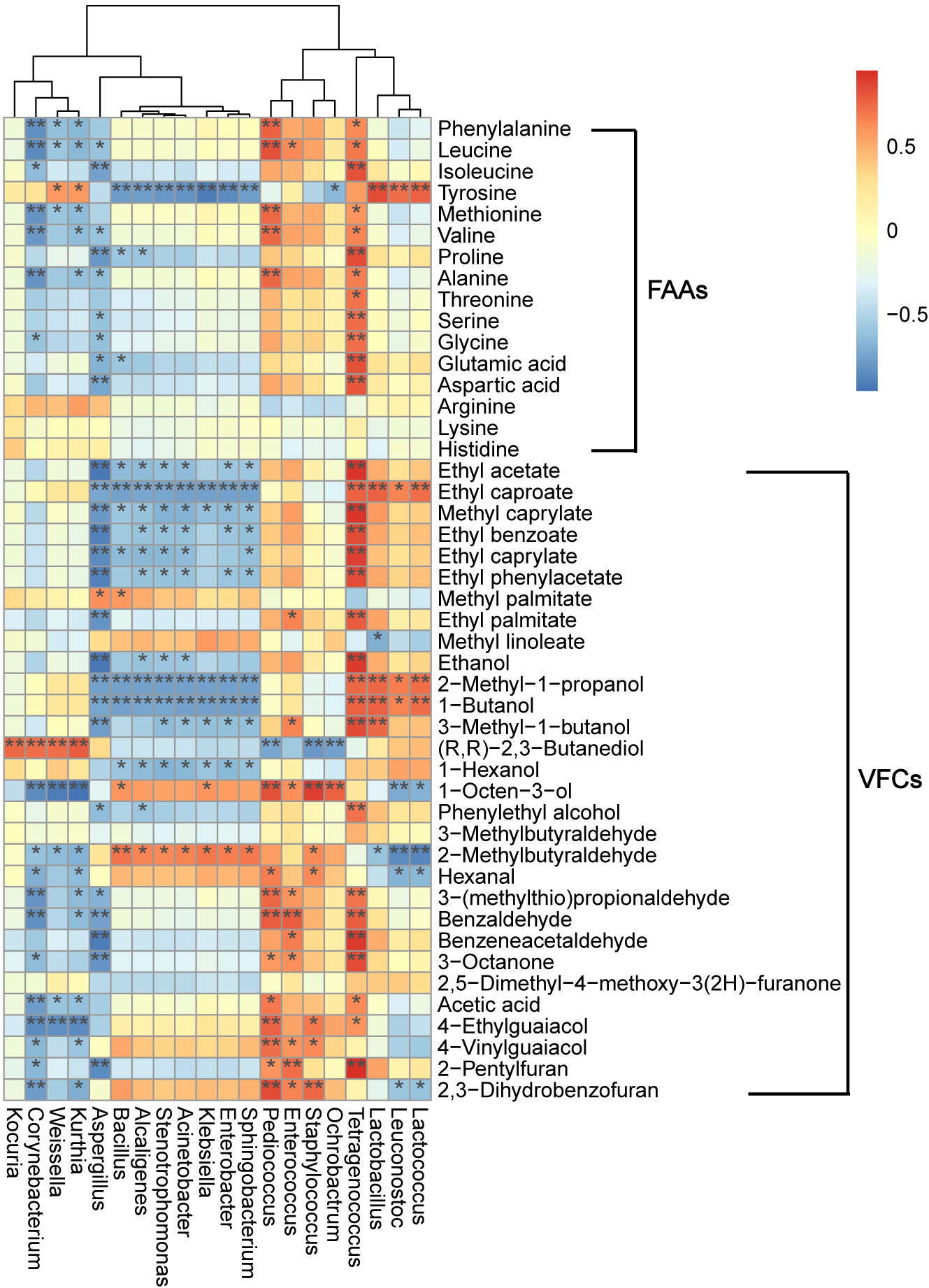

Supplement: SUPPLEMENTARY FIGURE S5 — Heatmap of correlations among microbial genera and metabolites. Metabolites included all FAAs and the 30 most abundant VFCs that were detected. The microorganisms comprised the 20 most abundant genera. Correlation strength (based on Spearman’s r-value) and correlation significance values are shown as shaded colors (red, positive correlation; blue, negative correlation). Heatmap values range from + 1.0 to −1.0. Values above/below zero represent positive/negative correlations, respectively, between genera and parameters. *p < 0.05, **p < 0.01. [file Image_5.TIF]

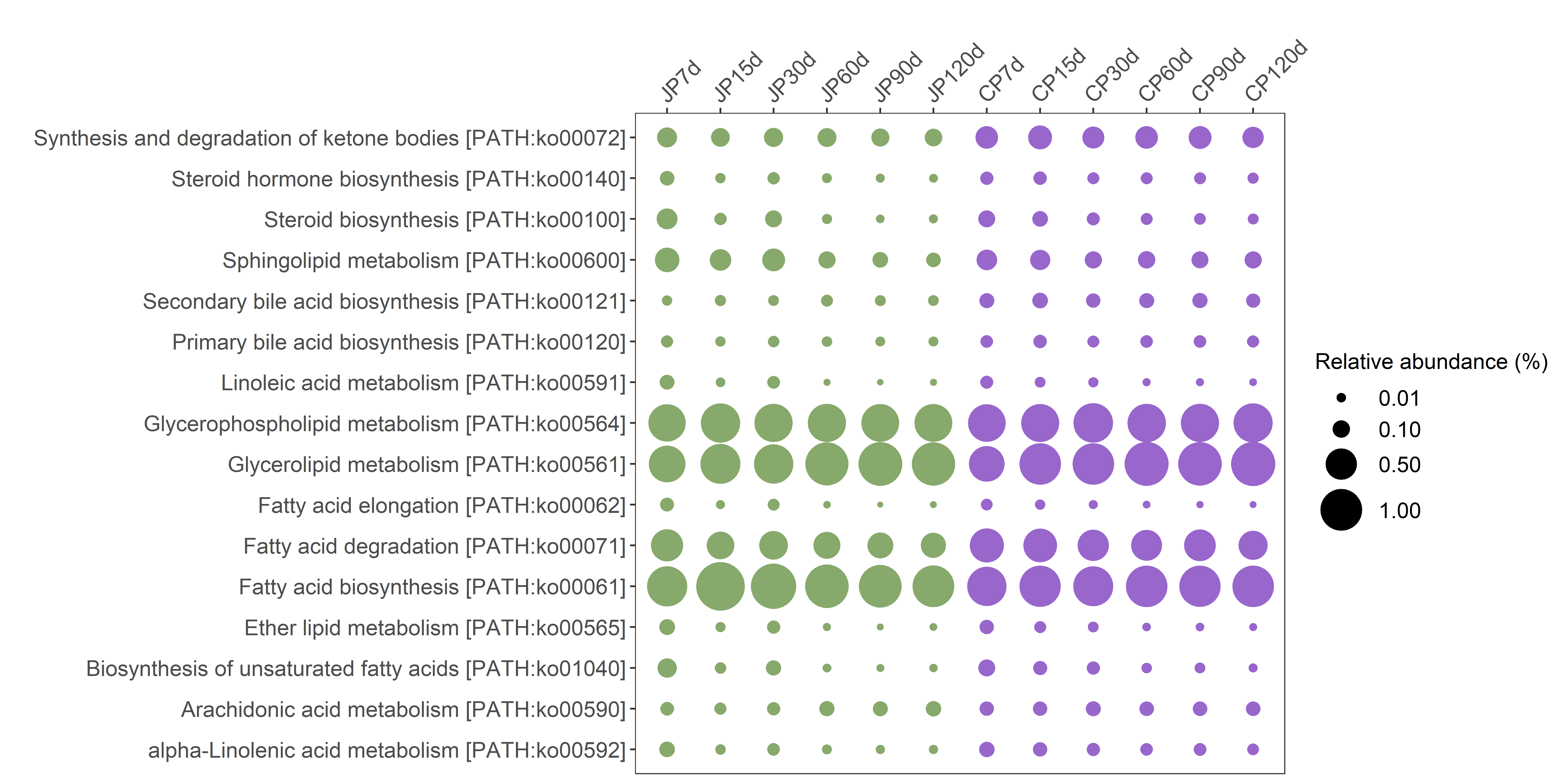

Supplement: SUPPLEMENTARY FIGURE S7 — Variation in the abundances of functional categories associated with lipid metabolism from soy sauce mash microbial communities based on metagenome annotations for two different fermentation processes. Functional classes were determined at the level 3 classifications of KEGG annotations using whole shotgun metagenome assembly-derived ORFs. JP, Japanese-type; CP, Cantonese-type; KEGG, Kyoto Encyclopedia of Genes and Genomes; ORFs, open reading frames. [file Image_7.TIF]
