# Supplementary material for: Comparative evaluation of the microbial diversity and metabolite profiles of Japanese-style and Cantonese-style soy sauce fermentation
Source: Front Microbiol. 2022 Aug 8;13:976206. doi: 10.3389/fmicb.2022.976206 (PMC9393507; doi:10.3389/fmicb.2022.976206)
Supplement: Supplementary file 8 [file Data_Sheet_1.docx]

Supplementary Tables for

**Comparative Evaluation of the Microbial Diversity and Metabolite Profiles of Japanese-style and Cantonese-style Soy Sauce Fermentation**

**Guiliang Tan^a^, Yi Wang^a^, Min Hu^b*^, Xueyan Li^a^, Xiangli Li^c^, Ziqiang Pan^a^, Mei Li^a^, Lin Li^a^, Ziyi Zheng^a^**

^a^ *School of Material Science and Food Engineering, University of Electronic Science and Technology of China, Zhongshan Institute, Zhongshan 528402, China;*

^b^ *School of Environmental and Safety Engineering, Changzhou University, Changzhou 213164, China*

^c^ *School of Health Industry, Zhongshan Torch Vocational and Technical College, Zhongshan 528436, China.*

^∗^*Correspondence to: Min Hu, School of Environmental and Safety Engineering, Changzhou University, Changzhou 213164, China. E-mail:* [*humin@soil.gd.cn*](mailto:humin@soil.gd.cn).

**Table S1.** Characteristics of FAAs within soy sauce mash samples.

| **FAAs** | **Concentration (g/kg)** | | | | | | | | | | | |
| --- | --- | --- | --- | --- | --- | --- | --- | --- | --- | --- | --- | --- |
|  | **JP7d** | **JP15d** | **JP30d** | **JP60d** | **JP90d** | **JP120d** | **CP7d** | **CP15d** | **CP30d** | **CP60d** | **CP90d** | **CP120d** |
| Phenylalanine | 6.22 ± 0.55 | 10.3 ± 1.01 | 11.95 ± 1.08 | 13.22 ± 0.42 | 12.46 ± 1.48 | 13.76 ± 1.05 | 3.74 ± 0.22 | 9.92 ± 0.83 | 15.22 ± 1.20 | 15.98 ±0.98 | 17.55 ± 1.21 | 15.94 ± 1.28 |
| Leucine | 8.46 ± 0.51 | 12.22 ± 1.36 | 14.56 ± 0.91 | 15.79 ± 2.19 | 15.26 ± 0.31 | 16.88 ± 0.87 | 4.31 ± 0.57 | 12.51 ± 0.82 | 15.96 ± 1.17 | 18.52 ± 1.25 | 20.21 ± 1.39 | 20.44 ± 1.45 |
| Isoleucine | 3.82 ± 1.23 | 7.30 ± 0.21 | 9.25 ± 1.45 | 10.6 ± 0.83 | 12.07 ± 1.24 | 13.35 ± 0.82 | 2.28 ± 0.19 | 6.27 ± 0.32 | 10.97 ± 0.99 | 12.06 ± 1.07 | 13.29 ± 0.73 | 11.68 ± 0.87 |
| Tyrosine | 3.29 ± 0.15 | 5.78 ± 0.38 | 5.61 ± 0.11 | 6.15 ± 0.27 | 6.32 ± 0.84 | 6.85 ± 0.29 | 1.94 ± 0.35 | 5.18 ± 0.94 | 3.65 ± 0.25 | 3.05 ± 0.14 | 1.99 ± 0.12 | 3.17 ± 0.26 |
| Methionine | 1.72 ± 0.50 | 2.85 ± 0.35 | 3.23 ± 0.14 | 3.32 ± 0.22 | 3.17 ± 0.19 | 3.49 ± 0.66 | 1.26 ± 0.10 | 2.50 ± 0.19 | 3.66 ± 0.35 | 3.93 ± 0.26 | 4.36 ± 0.31 | 4.07 ± 0.11 |
| Valine | 4.34 ± 0.28 | 7.44 ± 0.69 | 9.39 ± 0.26 | 11.51 ± 0.55 | 11.25 ± 0.30 | 12.39 ± 0.88 | 3.05 ± 0.31 | 6.50 ± 0.47 | 12.04 ± 1.16 | 13.48 ± 1.12 | 14.98 ± 1.36 | 12.41 ± 0.83 |
| Proline | 4.46 ± 0.10 | 6.83 ± 0.28 | 8.29 ± 0.74 | 9.62 ± 0.36 | 9.60 ± 0.54 | 10.43 ± 0.73 | 1.24 ± 0.04 | 3.48 ± 0.20 | 8.93 ± 0.58 | 9.33 ± 0.54 | 10.16 ± 1.03 | 8.31 ± 0.72 |
| Alanine | 4.06 ± 0.31 | 6.31 ± 0.39 | 7.67 ± 0.13 | 9.00 ± 0.67 | 8.82 ± 0.71 | 9.67 ± 0.21 | 2.26 ± 0.11 | 5.34 ± 0.61 | 9.16 ± 0.71 | 10.23 ± 1.01 | 12.15 ± 1.26 | 11.65 ± 1.54 |
| Threonine | 3.57 ± 0.64 | 5.66±0.10 | 6.98 ± 0.66 | 7.66 ± 0.70 | 7.54 ± 0.26 | 8.19 ± 0.18 | 2.14 ± 0.16 | 4.37 ± 0.39 | 8.05 ± 0.24 | 7.75 ± 0.55 | 8.41 ± 0.34 | 6.47 ± 0.50 |
| Serine | 6.1 ± 0.01 | 8.51 ± 1.27 | 10.2 ± 1.13 | 11.21 ± 1.27 | 10.7 ± 0.46 | 11.73 ± 1.23 | 3.28 ± 0.28 | 6.81 ± 0.44 | 11.51 ± 0.39 | 11.03 ± 1.02 | 12.21 ± 0.75 | 9.79 ± 0.62 |
| Glycine | 2.08 ± 0.09 | 3.55 ± 0.18 | 4.97 ± 0.45 | 5.65 ± 0.76 | 5.56 ± 0.28 | 6.16 ± 0.37 | 0.97 ± 0.06 | 2.70 ± 0.17 | 5.77 ± 0.36 | 5.70 ± 0.51 | 6.23 ± 0.62 | 5.23 ± 0.31 |
| Glutamic acid | 4.28 ± 0.13 | 6.00 ± 0.32 | 7.19 ± 0.38 | 7.74 ± 0.24 | 7.26 ± 0.13 | 7.78 ± 0.76 | 3.39 ± 0.23 | 4.48 ± 0.33 | 7.39 ± 0.43 | 6.83 ±0 .23 | 7.44 ± 0.47 | 6.07 ± 0.30 |
| Aspartic acid | 6.86 ± 0.45 | 11.82 ± 0.44 | 15.15 ± 1.02 | 19.03 ± 1.33 | 18.67 ± 0.67 | 21.17 ± 1.43 | 4.80 ± 0.45 | 8.10 ± 2.35 | 17.68 ± 1.45 | 19.51 ± 2.45 | 20.6 ± 1.45 | 16.0 ± 1.72 |
| Arginine | 13.45 ± 1.04 | 17.11 ± 0.25 | 18.14 ± 1.26 | 10.03 ± 0.75 | 8.62 ± 0.63 | 8.69 ± 0.68 | 7.56 ± 0.66 | 14.43 ± 1.09 | 18.43 ± 1.31 | 8.59 ± 0.31 | 4.81 ± 0.34 | 3.77 ± 0.10 |
| Lysine | 6.75 ± 0.53 | 11.48 ± 0.23 | 13.04 ± 0.99 | 9.15 ± 0.39 | 8.32 ± 0.70 | 8.95 ± 0.43 | 3.79 ± 0.18 | 7.43 ± 0.63 | 14.54 ± 1.52 | 10.36 ± 0.53 | 9.79 ± 0.67 | 6.62 ± 0.56 |
| Histidine | 6.61 ± 0.21 | 8.82 ± 0.63 | 10.31 ± 0.81 | 6.78 ± 0.92 | 6.14 ± 0.59 | 6.59 ± 0.60 | 2.79 ± 0.12 | 4.98 ± 0.21 | 9.99 ± 0.21 | 8.18 ± 0.36 | 7.10 ± 0.61 | 5.11 ± 0.22 |

The concentration of each compound is shown as g/kg (dry weight).

Values reprent means ± SD (n=3). The concentration of each compound is represented by g/kg (dry weight).

**Table S2.** Differential analysis of metabolites in 120 d mash samples when comparing JP and CP fermentations (based on *t*-tests).

| **Category** | **Chemical compound** | ***P* value** |
| --- | --- | --- |
| FAAs | Phenylalanine | 0.08 |
|  | Leucine | 0.02 |
|  | Isoleucine | 0.07 |
|  | Tyrosine | 0.00 |
|  | Methionine | 0.21 |
|  | Valine | 0.98 |
|  | Proline | 0.02 |
|  | Alanine | 0.09 |
|  | Threonine | 0.00 |
|  | Serine | 0.07 |
|  | Glycine | 0.03 |
|  | Glutamic acid | 0.02 |
|  | Aspartic acid | 0.02 |
|  | Arginine | 0.00 |
|  | Lysine | 0.00 |
|  | Histidine | 0.02 |
| VFCs | Ethyl acetate | 0.00 |
|  | Ethyl caproate | 0.00 |
|  | Methyl caprylate | 0.01 |
|  | Ethyl benzoate | 0.00 |
|  | Ethyl caprylate | 0.00 |
|  | Ethyl phenylacetate | 0.00 |
|  | Methyl palmitate | 0.07 |
|  | Ethyl palmitate | 0.00 |
|  | Methyl linoleate | 0.00 |
|  | Ethanol | 0.00 |
|  | 2-Methyl-1-propanol | 0.00 |
|  | 1-Butanol | 0.00 |
|  | 3-Methyl-1-butanol | 0.00 |
|  | (R,R)-2,3-Butanediol | 0.00 |
|  | 1-Hexanol | 0.00 |
|  | 1-Octen-3-ol | 0.00 |
|  | Phenylethyl alcohol | 0.00 |
|  | 3-Methylbutyraldehyde | 0.00 |
|  | 2-Methylbutyraldehyde | 0.02 |
|  | Hexanal | 0.00 |
|  | 3-(methylthio)propionaldehyde | 0.01 |
|  | Benzaldehyde | 0.02 |
|  | Benzeneacetaldehyde | 0.00 |
|  | 3-Octanone | 0.02 |
|  | 2,5-Dimethyl-4-methoxy-3(2H)-furanone | 0.00 |
|  | Acetic acid | 0.00 |
|  | 4-Ethylguaiacol | 0.18 |
|  | 4-Vinylguaiacol | 0.00 |
|  | 2-Pentylfuran | 0.01 |
|  | 2,3-Dihydrobenzofuran | 0.00 |

**Table S3.** Characteristics of VFCs from mash samples.

|  |  | | | |  |  | **Average concentration (μg/kg)** ^a^ | | | |  |  |  |
| --- | --- | --- | --- | --- | --- | --- | --- | --- | --- | --- | --- | --- | --- |
|  | **RT** ^b^ | **JP7d** | **JP15d** | **JP30d** | **JP60d** | **JP90d** | **JP120d** | **CP7d** | **CP15d** | **CP30d** | **CP60d** | **CP90d** | **CP120d** |
| **Esters (32)** |  |  |  |  |  |  |  |  |  |  |  |  |  |
| Methyl acetate | 1.8606 | 19.12 | 18.68 | 10.25 | / | / | / | / | / | / | / | / | / |
| Ethyl acetate | 2.4567 | 11.36 | 41.55 | 23.24 | 176.93 | 238.75 | 240.65 | 4.73 | 18.68 | 79.24 | 133.94 | 145.17 | 136.98 |
| Methyl butyrate | 4.0723 | 6.05 | 5.96 | 5.00 | / | / | / | 8.13 | 6.92 | 7.85 | 4.79 | 6.75 | 4.98 |
| Methyl 2-methylbutyrate | 5.3356 | 2.83 | / | 1.85 | / | / | / | 2.02 | / | 4.81 | / | 10.16 | / |
| Ethyl butyrate | 5.9843 | / | / | / | 7.13 | 8.17 | 8.28 | / | / | 8.56 | 5.88 | 23.05 | / |
| Ethyl L(-)-lactate | 6.3553 | / | / | / | / | / | 83.47 | / | / | / | / | / | / |
| Isobutyl lactate | 6.3591 | / | / | / | 70.93 | 78.50 | / | / | / | / | / | / | / |
| Ethyl 2-methylbutyrate | 7.39 | / | / | / | 9.30 |  | 19.00 | / | / | 2.88 | 9.17 | 8.26 | 3.86 |
| Ethyl isovalerate | 7.4912 | / | / | / | 9.63 | 17.84 | 23.82 | / | / | / | 8.35 | / | 4.33 |
| Isoamyl acetate | 8.2072 | / | / | / | 11.15 | 17.15 | 18.82 | / | / | / | 9.83 | / | / |
| Ethyl valerate | 8.9494 | / | / | / | 6.35 | 8.11 | 9.37 | / | / | / | / | 12.37 | / |
| Methyl hexanoate | 9.6803 | 5.24 | 8.16 | 9.71 | 6.99 | 6.08 | 8.70 | / | / | 3.28 | 5.25 | 3.25 | 4.57 |
| Ethyl caproate | 11.6934 | / | / | / | 111.74 | 141.84 | 150.94 | / | / | / | / | / | / |
| Methyl heptanoate | 12.2519 | 2.62 | 4.22 | 4.76 | 5.35 | 6.53 | 9.89 | / | / | 3.07 | 4.04 | 2.45 | 2.41 |
| Isopentyl lactate | 13.1816 | / | / | / | 9.07 | 11.71 | 11.50 | / | / | / | / | / | / |
| Ethyl heptanoate | 13.7589 | / | / | / | 25.12 | 31.11 | 30.44 | / | / | / | / | / | / |
| Methyl caprylate | 14.2386 | 9.84 | 16.55 | 19.94 | 26.75 | 37.31 | 35.90 | 5.72 | 11.46 | 19.99 | 29.02 | 15.23 | 24.89 |
| Ethyl benzoate | 15.0785 | / | / | / | 53.18 | 72.60 | 82.94 | / | / | / | 8.50 | 7.59 | 7.19 |
| Diethyl succinate | 15.2134 | / | / | / | 19.62 | 39.95 | 46.07 |  |  |  |  |  |  |
| Ethyl caprylate | 15.4757 | 1.93 | 2.68 | 2.56 | 72.87 | 93.72 | 107.38 | / | / | 4.96 | 7.52 | 6.50 | 4.61 |
| Methyl nonanoate | 15.9105 | 1.61 | 2.31 | 2.45 | 5.63 | 6.91 | 6.96 | / | / | / | 2.08 | / | / |
| Ethyl phenylacetate | 16.2668 | / | / | / | 38.22 | 59.15 | 69.84 | / | / | / | 7.06 | 5.42 | 1.87 |
| Ethyl nonanoate | 16.994 | / | / | / | 9.81 | 12.88 | 15.04 | / | / | / | / | / | / |
| gamma-Nonanolactone | 17.9873 | 1.60 | 3.96 | 4.82 | 18.88 | 23.99 | 28.27 | / | / | 1.97 | 2.48 | 1.80 | 2.76 |
| Ethyl caprate | 18.366 | / | / | / | 8.72 | 10.89 | 11.83 | / | / | / | / | / | / |
| Ethyl laurate | 20.8514 | / | / | / | 6.00 | 6.92 | 8.39 | / | / | / | / | / | / |
| methyl tetradecanoate | 22.3319 | 2.09 | 2.50 | 2.41 | 1.83 | 2.06 | 2.41 | 3.13 | 3.81 | 2.32 | 3.94 | 1.48 | 1.73 |
| Ethyl tetradecanoate | 23.0706 | / | / | / | 5.24 | 7.36 | 9.49 |  |  |  |  |  |  |
| Methyl palmitate | 24.4312 | 54.13 | 72.26 | 65.90 | 41.35 | 41.66 | 44.04 | 63.29 | 90.96 | 63.12 | 74.32 | 40.03 | 50.43 |
| Ethyl palmitate | 25.0872 | 6.74 | 7.64 | 7.57 | 58.07 | 87.41 | 110.76 | 8.91 | 13.42 | 12.37 | 18.68 | 12.53 | 11.09 |
| Methyl linoleate | 26.0619 | 16.60 | 26.26 | 22.18 | 9.03 | 11.25 | 12.71 | 34.21 | / | 31.88 | 37.51 | 17.68 | 25.91 |
| Ethyl linoleate | 26.6655 | 0 | / | / | 23.26 | 40.29 | 46.61 | / | / | / | 4.77 | 4.17 | / |
| **subtotal** |  | **141.77** | **212.73** | **182.66** | **848.17** | **1120.12** | **1253.51** | **130.14** | **145.25** | **246.30** | **377.13** | **323.89** | **287.61** |
| **Alcohols (16)** |  |  |  |  |  |  |  |  |  |  |  |  |  |
| Ethanol | 1.617 | 29.30 | 52.24 | 37.66 | 355.03 | 366.80 | 398.43 | 23.12 | 49.78 | 65.91 | 90.61 | 90.62 | 86.61 |
| 3-Methoxy-1-propanol | 2.0331 | / | / | / | / | / | 61.40 | / | / | / | / | / | / |
| 2-Methyl-1-propanol | 2.6255 | / | / | / | 43.93 | 51.78 | 51.72 | / | / | / | / | / | / |
| 1-Butanol | 3.124 | / | / | / | 144.12 | 150.25 | 165.87 | / | / | / | / | / | / |
| 3-Methyl-1-butanol | 4.3235 | 26.26 | 27.05 | 23.88 | 124.24 | 158.83 | 100.40 | 17.26 | 27.90 | 29.47 | 39.49 | 23.14 | 38.90 |
| 2-Methyl-1-butanol | 4.451 | / | / | / | / | / | 59.21 | / | 10.95 | / | / | 14.81 | 0.00 |
| 1-Pentanol | 5.107 | 4.67 | 4.78 | 3.12 | / | / | / | / | / | 4.51 | 6.88 | 5.59 | 5.99 |
| (R,R)-2,3-Butanediol | 5.598 | 30.91 | 36.10 | 5.68 | / | / | 20.74 | / | / | / | / | / | / |
| 2,3-Butanediol | 5.8379 | 10.99 | 7.94 | 24.67 | 29.70 | 11.35 | 27.39 | / | / | 3.35 | / | / | 0.00 |
| Furfuryl alcohol | 7.5737 | / |  |  | 17.23 | 37.30 | 56.60 | / | / | / | 8.84 | / | 0.00 |
| 1-Hexanol | 7.9671 | / | 44.57 | 29.61 | 29.30 | 32.20 | 36.91 | 4.35 | / | / | 34.15 | 14.60 | 15.02 |
| 1-Octen-3-ol | 11.191 | 56.22 | 62.77 | 51.39 | 90.34 | 110.62 | 122.10 | 131.48 | 177.38 | 238.04 | 316.00 | 257.58 | 297.41 |
| 3-Octanol | 11.5809 | 1.53 | 2.42 | 1.93 | 5.91 | 7.10 | 8.93 | 3.58 | 4.34 | 7.34 | 8.49 | 5.85 | 6.59 |
| trans-2-Octen-1-ol | 13.1665 | 2.63 | 3.13 | / | / | / | / | / | 4.01 | 5.01 | / | 5.07 | 0.00 |
| Phenylethyl alcohol | 14.0512 | 6.67 | 8.37 | 9.71 | 156.10 | 221.67 | 269.20 | 4.30 | 11.16 | 18.93 | 37.95 | 29.33 | 0.00 |
| 2-Ethyl-2-phenylethanol | 16.4055 | / | / | / | 18.48 | 22.85 | 27.21 | / | / | / | / | / | / |
| **subtotal** |  | **169.18** | **249.38** | **187.66** | **1014.37** | **1170.75** | **1406.12** | **184.09** | **285.52** | **372.56** | **542.41** | **446.59** | **450.52** |
| **Aldehydes (10)** |  |  |  |  |  |  |  |  |  |  |  |  |  |
| Isobutyraldehyde | 2.0256 | / | / | / | / | / | / | / | / | 14.09 | 18.89 | 16.75 | 14.61 |
| 3-Methylbutyraldehyde | 2.8953 | 11.16 | 17.24 | 14.39 | 23.36 | 28.08 | 30.85 | 10.59 | 21.16 | 57.04 | 61.70 | / | / |
| 2-Methylbutyraldehyde | 3.0302 | 3.17 | 10.60 | 8.54 | / | / | / | 6.46 | 13.74 | 36.72 | 44.24 | 33.77 | 34.75 |
| 3-Methyl-2-butenal | 5.4894 | / | / | / | / | / | / | / | / | / | 14.58 | 8.50 | 7.62 |
| Hexanal | 5.8904 | / | 4.52 | 3.20 | / | / | / | / | 4.12 | 10.08 | 15.25 | 16.29 | 16.95 |
| Furfural | 6.8315 | / | / | / | 21.20 | 31.62 | 39.27 | / | / | / | 12.58 | 5.55 | 5.66 |
| 3-(methylthio)propionaldehyde | 9.0394 | / | / | 2.36 | 10.96 | 14.08 | 20.85 | / | / | 8.47 | 23.66 | 24.74 | 30.83 |
| Benzaldehyde | 10.6212 | 6.65 | 13.31 | 14.04 | 43.13 | 54.78 | 69.84 | 6.70 | 22.64 | 34.77 | 94.19 | 50.97 | 90.10 |
| Benzeneacetaldehyde | 12.6492 | 9.91 | 14.42 | 13.54 | 53.78 | 71.97 | 94.41 | 14.45 | 29.70 | 37.14 | 51.70 | 37.18 | 49.02 |
| 2-Phenyl-2-butenal | 16.7091 | / | / | / | 4.68 | 12.33 | 11.41 | / | / | / | 3.42 | / | / |
| **subtotal** |  | **30.89** | **60.09** | **56.08** | **157.11** | **212.86** | **266.63** | **38.20** | **91.36** | **198.31** | **340.21** | **193.75** | **249.54** |
| **Acids (2)** |  |  |  |  |  |  |  |  |  |  |  |  |  |
| Acetic acid | 2.6216 | 14.74 | / | 14.23 | 98.75 | 96.79 | 122.49 | / | / | 112.11 | 158.88 | 210.49 | 258.10 |
| Octanoic acid | 15.0746 | / | / | / | / | / | / | / | / | 3.91 | / | 19.90 | / |
| **subtotal** |  | **14.74** | **0.00** | **14.23** | **98.75** | **96.79** | **122.49** | **0.00** | **0.00** | **116.02** | **158.88** | **230.39** | **258.10** |
| **Phenols (4)** |  |  |  |  |  |  |  |  |  |  |  |  |  |
| Guaiacol | 13.5865 | / | / | / | / | / | / | / | 3.80 | 5.18 | 9.24 | 6.01 | 8.35 |
| 4-Ethylphenol | 14.9584 | / | / | / | / | / | / | / | / | 15.06 | 41.39 | 13.27 | / |
| 4-Ethylguaiacol | 16.7916 | / | / | 1.47 | 7.99 | 10.48 | 14.21 | 2.09 | 2.23 | 42.07 | 119.11 | 54.36 | 12.91 |
| 4-Vinylguaiacol | 17.305 | 15.70 | 53.01 | 75.66 | 75.63 | 35.45 | 23.30 | 28.36 | 88.69 | 82.11 | 102.17 | 65.08 | 81.04 |
| **subtotal** |  | **15.70** | **53.01** | **77.13** | **83.62** | **45.92** | **37.51** | **30.45** | **94.72** | **144.42** | **271.91** | **138.72** | **102.30** |
| **Others (21)** |  |  |  |  |  |  |  |  |  |  |  |  |  |
| Acetone | 1.737 | / | / | / | / | / | / | / | / | 22.24 | 28.23 | / | / |
| Dimethyl ether | 2.0218 | / | / | / | / | 43.57 | / | / | / | / | / | / | / |
| 3-Aminopyrrolidine | 2.2805 | 25.51 | / | / | / | / | / | / | / | / | / | / | / |
| 2-Butanone | 2.2994 | / | / | / | / | / | / | 24.02 | 16.16 | / | 36.86 | / | / |
| Hexane | 2.303 | / | 30.26 | 18.75 |  | / | / | / | / | / | / | / | / |
| 2-Pentanone | 3.3976 | 7.96 | 7.92 | 6.57 | / | / | / | 11.12 | 11.04 | 11.84 | 10.94 | 8.92 | 10.51 |
| 3-Pentanone | 3.5625 | 6.95 | 8.93 | 7.05 | / | / | / | 10.32 | 9.96 | 10.73 | 12.01 | / | / |
| 3-Hydroxy-2-butanone | 3.8813 | / | / | / | 9.24 | 9.24 | 9.85 | / | / | 3.09 | / | / | / |
| 2-Methylpyrazine | 6.6064 | / | 6.33 | 6.17 | 9.14 | 12.05 | 14.11 | / | / | / | / | / | / |
| 2-Heptanone | 8.6007 | / | / | / | / | / | / | / | / | 5.47 | 7.45 | 4.65 | 9.19 |
| n-Nonane | 8.8556 | / | 2.66 | 2.95 |  | 2.32 | / | 3.61 | 3.85 | 3.35 | 5.42 | 4.72 | 6.22 |
| 3,5,5-Trimethyl-1-hexene | 11.0374 | / | / | / | / | / | / | / | / | / | 6.10 | 3.45 | 13.1703 |
| 3-Octanone | 11.3673 | / | / | 3.14 | 11.12 | 13.63 | 15.17 | 4.57 | 4.99 | 8.82 | 13.39 | 11.43 | 9.72 |
| 2-Pentylfuran | 11.4684 | / | 3.00 | 3.41 | 10.97 | 16.59 | 20.57 | 2.39 | 4.05 | 8.71 | 11.00 | 8.08 | 11.05 |
| Decane | 11.6633 | 10.82 | 11.43 | 11.26 | / | / | / | 13.85 | 15.50 | 16.46 | 18.55 | 18.29 | 16.93 |
| Undecane | 13.7739 | / | / | 1.87 | / | / | / | 2.16 | 2.85 | 3.85 | 4.18 | 4.21 | 4.12 |
| 3-Hydroxy-2-methyl-4-pyrone | 14.0513 | / | / | / | / | / | / | / | / | / | / | / | 30.86 |
| 4-Methoxy-2,5-dimethyl-3(2H)-furanone | 14.5797 | / | / | / | / | 32.90 | / | / | / | / | / | / | / |
| 4-Chloroanisole | 14.7148 | / | / | / | 45.79 | 59.57 | / |  |  |  |  |  |  |
| 2,3-Dihydrobenzofuran | 15.7906 | 2.29 | 5.78 | 7.85 | 9.42 | 5.36 | / | 4.25 | 23.52 | 22.86 | 25.30 | 10.97 | 24.20 |
| 1,3-Di-tert-butylbenzene | 16.4166 | 3.60 | 2.51 | / | / | / | / | 3.09 | 5.63 | 3.36 | 7.77 | 4.43 | 5.17 |
| **subtotal** |  | **5.89** | **8.29** | **7.85** | **9.42** | **5.36** | **0.00** | **7.34** | **29.15** | **26.22** | **33.07** | **15.40** | **29.37** |

^a^ The concentration of each compound is represented by μg/kg (dry weight).

^b^ RT: retention time.

**Table. S4.** Differential analysis of metabolic pathways at the KEGG level 3 category in mash samples (60 to 120 d of fermentation) when comparing JP and CP fermentations (based on *t*-tests).

| **Category** | ***P* value** |
| --- | --- |
| **Carbohydrate metabolism** |  |
| Amino sugar and nucleotide sugar metabolism [PATH:ko00520] | 0.01 |
| Ascorbate and aldarate metabolism [PATH:ko00053] | 0.02 |
| Butanoate metabolism [PATH:ko00650] | 0.00 |
| C5-Branched dibasic acid metabolism [PATH:ko00660] | 0.01 |
| Citrate cycle (TCA cycle) [PATH:ko00020] | 0.01 |
| Fructose and mannose metabolism [PATH:ko00051] | 0.08 |
| Galactose metabolism [PATH:ko00052] | 0.05 |
| Glycolysis / Gluconeogenesis [PATH:ko00010] | 0.01 |
| Glyoxylate and dicarboxylate metabolism [PATH:ko00630] | 0.00 |
| Inositol phosphate metabolism [PATH:ko00562] | 0.00 |
| Pentose and glucuronate interconversions [PATH:ko00040] | 0.00 |
| Pentose phosphate pathway [PATH:ko00030] | 0.02 |
| Propanoate metabolism [PATH:ko00640] | 0.01 |
| Pyruvate metabolism [PATH:ko00620] | 0.00 |
| Starch and sucrose metabolism [PATH:ko00500] | 0.01 |
| **Amino acid metabolism** |  |
| Alanine, aspartate and glutamate metabolism [PATH:ko00250] | 0.07 |
| Arginine and proline metabolism [PATH:ko00330] | 0.02 |
| Arginine biosynthesis [PATH:ko00220] | 0.00 |
| Cysteine and methionine metabolism [PATH:ko00270] | 0.25 |
| Glycine, serine and threonine metabolism [PATH:ko00260] | 0.52 |
| Histidine metabolism [PATH:ko00340] | 0.00 |
| Lysine biosynthesis [PATH:ko00300] | 0.00 |
| Lysine degradation [PATH:ko00310] | 0.00 |
| Phenylalanine metabolism [PATH:ko00360] | 0.02 |
| Phenylalanine, tyrosine and tryptophan biosynthesis [PATH:ko00400] | 0.08 |
| Tryptophan metabolism [PATH:ko00380] | 0.01 |
| Tyrosine metabolism [PATH:ko00350] | 0.03 |
| Valine, leucine and isoleucine biosynthesis [PATH:ko00290] | 0.01 |
| Valine, leucine and isoleucine degradation [PATH:ko00280] | 0.01 |
| **Lipid metabolism** |  |
| alpha-Linolenic acid metabolism [PATH:ko00592] | 0.00 |
| Arachidonic acid metabolism [PATH:ko00590] | 0.03 |
| Biosynthesis of unsaturated fatty acids [PATH:ko01040] | 0.04 |
| Ether lipid metabolism [PATH:ko00565] | 0.10 |
| Fatty acid biosynthesis [PATH:ko00061] | 0.11 |
| Fatty acid degradation [PATH:ko00071] | 0.01 |
| Fatty acid elongation [PATH:ko00062] | 0.44 |
| Glycerolipid metabolism [PATH:ko00561] | 0.12 |
| Glycerophospholipid metabolism [PATH:ko00564] | 0.10 |
| Linoleic acid metabolism [PATH:ko00591] | 0.01 |
| Primary bile acid biosynthesis [PATH:ko00120] | 0.00 |
| Secondary bile acid biosynthesis [PATH:ko00121] | 0.00 |
| Sphingolipid metabolism [PATH:ko00600] | 0.15 |
| Steroid biosynthesis [PATH:ko00100] | 0.01 |
| Steroid hormone biosynthesis [PATH:ko00140] | 0.00 |
| Synthesis and degradation of ketone bodies [PATH:ko00072] | 0.00 |

**Table S5.** Selected enzymes and their relative abundances that are involved in the metabolism of substrates associated with the generation of flavoring compounds.

| **Product or substrate** | **EC number of enzymes** | **Enzyme name** | **Pathway in KEGG** | **Relative abundances (Transcripts per million, TPM)** | | | | | | | | | | | |
| --- | --- | --- | --- | --- | --- | --- | --- | --- | --- | --- | --- | --- | --- | --- | --- |
|  |  |  |  | **JP7d** | **JP15d** | **JP30d** | **JP60d** | **JP90d** | **JP120d** | **CP7d** | **CP15d** | **CP30d** | **CP60d** | **CP90d** | **CP120d** |
| Ethanol | 1.1.1.1 | alcohol dehydrogenase | ko00620 | 760.10 | 1104.73 | 1059.59 | 1183.95 | 1186.31 | 1140.21 | 771.06 | 719.66 | 781.43 | 845.84 | 816.27 | 890.48 |
|  | 1.1.1.2 | alcohol dehydrogenase (NADP+) | ko00620 | 120.90 | 23.71 | 69.84 | 1.53 | 0.79 | 1.55 | 52.81 | 33.87 | 16.32 | 4.78 | 2.63 | 2.57 |
|  | 1.1.2.8 | alcohol dehydrogenase (cytochrome c) | ko00620 | 0.59 | 0.04 | 0.19 | 0.00 | 0.13 | 0.08 | 1.27 | 0.98 | 0.73 | 0.41 | 1.42 | 0.00 |
| Acetate | 2.7.2.1 | acetate kinase | ko00620 | 269.74 | 676.13 | 548.74 | 541.95 | 505.27 | 452.74 | 288.38 | 374.88 | 309.18 | 410.42 | 375.57 | 427.93 |
|  | 3.6.1.7 | acylphosphatase | ko00620 | 262.39 | 361.72 | 280.53 | 355.79 | 371.95 | 375.95 | 101.91 | 102.90 | 167.77 | 211.77 | 169.34 | 236.32 |
|  | 2.8.3.1 | propionate CoA-transferase | ko00620 | 15.68 | 64.35 | 47.36 | 30.36 | 22.73 | 12.88 | 15.26 | 11.06 | 10.24 | 10.08 | 8.46 | 6.27 |
|  | 6.2.1.13 | acetate---CoA ligase (ADP-forming) subunit alpha | ko00620 | 0.00 | 0.06 | 0.15 | 0.00 | 0.00 | 0.00 | 13.20 | 1.85 | 1.35 | 0.01 | 0.44 | 0.12 |
|  | 6.2.1.1 | acetyl-CoA synthetase | ko00620 | 177.29 | 90.66 | 99.59 | 146.98 | 102.70 | 79.38 | 333.13 | 513.48 | 368.61 | 383.28 | 463.93 | 294.93 |
|  | 1.2.1.10 | acetaldehyde dehydrogenase | ko00620 | 121.18 | 429.71 | 338.67 | 413.78 | 450.15 | 435.15 | 162.25 | 110.08 | 184.44 | 205.58 | 201.18 | 238.51 |
|  | 1.2.1.3 | aldehyde dehydrogenase (NAD+) | ko00620 | 247.05 | 169.00 | 215.34 | 425.34 | 402.32 | 387.13 | 533.48 | 816.73 | 630.60 | 739.90 | 812.21 | 626.61 |
|  | 1.13.12.4 | lactate 2-monooxygenase | ko00620 | 20.27 | 2.02 | 9.22 | 0.00 | 0.00 | 0.12 | 7.93 | 5.96 | 2.90 | 0.56 | 0.64 | 0.18 |
| Glutamate | 1.4.1.13 | glutamate synthase (NADPH) large chain | ko00250 | 168.56 | 140.02 | 141.20 | 415.78 | 435.68 | 462.57 | 426.21 | 503.59 | 527.72 | 545.69 | 585.55 | 482.15 |
|  | 6.3.1.2 | glutamine synthetase | ko00250 | 436.05 | 490.88 | 489.98 | 606.24 | 643.30 | 630.08 | 463.99 | 548.70 | 530.09 | 580.91 | 574.49 | 576.42 |
|  | 1.4.1.14 | glutamate synthase (NADH) | ko00250 | 27.04 | 7.87 | 19.15 | 4.87 | 0.45 | 5.07 | 19.69 | 11.67 | 5.12 | 3.55 | 2.78 | 0.62 |
|  | 3.5.1.2 | glutaminase | ko00250 | 43.56 | 18.71 | 17.00 | 11.78 | 12.13 | 10.19 | 86.85 | 64.30 | 37.81 | 30.61 | 35.32 | 27.49 |
| Arginine | 3.5.3.6 | arginine deiminase | ko00220 | 180.84 | 463.09 | 362.04 | 371.38 | 377.65 | 360.03 | 107.30 | 113.54 | 165.39 | 193.15 | 171.08 | 240.39 |
| Pyruvate | 1.2.3.3 | pyruvate oxidase | ko00620 | 193.00 | 701.06 | 547.31 | 539.83 | 491.91 | 438.27 | 210.66 | 341.00 | 282.47 | 380.93 | 363.26 | 393.01 |
|  | 4.1.1.1 | pyruvate decarboxylase | ko00620 | 104.62 | 17.79 | 68.97 | 5.29 | 1.32 | 6.40 | 85.47 | 42.00 | 17.29 | 8.42 | 7.88 | 2.26 |
|  | 1.2.7.11 | 2-oxoacid oxidoreductase | ko00620 | 28.91 | 61.07 | 60.89 | 106.45 | 74.50 | 57.37 | 103.38 | 288.23 | 194.01 | 272.05 | 306.30 | 205.69 |
|  | 1.2.7.1 | pyruvate ferredoxin oxidoreductase alpha subunit | ko00620 | 11.53 | 14.06 | 29.52 | 188.35 | 240.44 | 256.18 | 47.00 | 31.18 | 119.60 | 116.29 | 115.87 | 147.45 |
| Lactate | 1.1.1.27 | L-lactate dehydrogenase | ko00620 | 296.22 | 787.15 | 662.99 | 945.82 | 997.37 | 978.66 | 233.89 | 419.73 | 525.24 | 667.25 | 604.75 | 711.10 |
|  | 1.1.1.28 | D-lactate dehydrogenase | ko00620 | 265.07 | 827.90 | 635.55 | 482.18 | 381.96 | 301.88 | 312.72 | 459.27 | 285.24 | 374.43 | 385.41 | 342.78 |
| Histidine | 4.1.1.22 | histidine decarboxylase | ko00340 | 0.00 | 0.06 | 0.08 | 2.12 | 1.80 | 3.97 | 4.59 | 2.70 | 4.82 | 2.02 | 2.71 | 3.01 |
| Alanine，aspartate | 4.1.1.12 | aspartate 4-decarboxylase | ko00250 | 0.47 | 1.54 | 6.77 | 40.43 | 52.85 | 61.66 | 3.07 | 7.29 | 43.35 | 49.67 | 49.14 | 67.43 |
|  | 2.6.1.1 | aspartate aminotransferase | ko00250 | 218.51 | 42.49 | 115.55 | 14.80 | 5.75 | 8.13 | 237.15 | 123.33 | 97.50 | 44.11 | 54.32 | 29.34 |
|  | 2.6.1.2 | alanine transaminase | ko00250 | 67.25 | 32.49 | 42.67 | 193.06 | 246.77 | 253.44 | 96.79 | 52.30 | 123.82 | 121.18 | 118.62 | 144.10 |
|  | 1.4.1.1 | alanine dehydrogenase | ko00250 | 78.21 | 52.43 | 98.49 | 387.39 | 462.79 | 548.80 | 110.57 | 265.58 | 354.16 | 443.97 | 413.88 | 469.78 |
|  | 2.6.1.44 | alanine-glyoxylate transaminase | ko00250 | 28.05 | 4.16 | 10.78 | 5.33 | 1.25 | 1.20 | 9.37 | 7.72 | 3.83 | 3.08 | 6.17 | 0.94 |
| Hexadecanoate | 3.1.2.22 | palmitoyl-protein thioesterase | ko00062 | 10.38 | 2.47 | 9.34 | 0.07 | 0.00 | 0.00 | 10.26 | 6.35 | 2.56 | 0.79 | 1.10 | 0.18 |
| Phenylethyl alcohol | 1.1.1.90 | aryl-alcohol dehydrogenase | ko00360 | 23.63 | 34.75 | 27.75 | 30.07 | 25.75 | 35.35 | 16.11 | 14.60 | 13.80 | 11.13 | 9.01 | 8.25 |
|  | 1.11.1.21 | catalase-peroxidase | ko00360 | 36.31 | 11.85 | 16.75 | 5.08 | 2.27 | 2.61 | 62.34 | 25.00 | 33.11 | 13.73 | 19.96 | 10.10 |
|  | 3.5.1.4 | amidase | ko00360 | 567.33 | 199.19 | 359.44 | 239.69 | 266.99 | 302.45 | 351.69 | 231.59 | 206.36 | 165.01 | 161.80 | 166.14 |
|  | 1.2.1.39 | phenylacetaldehyde dehydrogenase | ko00360 | 4.46 | 1.56 | 1.04 | 1.72 | 0.74 | 1.15 | 68.30 | 16.93 | 23.49 | 9.50 | 16.97 | 9.01 |
|  | 4.1.1.28 | aromatic-L-amino-acid decarboxylase | ko00360 | 60.76 | 12.83 | 39.58 | 0.05 | 0.00 | 0.13 | 41.09 | 26.41 | 13.01 | 5.31 | 2.54 | 2.80 |
|  | 1.4.3.4 | monoamine oxidase | ko00360 | 147.52 | 25.22 | 83.70 | 1.61 | 1.33 | 2.25 | 99.50 | 64.82 | 30.71 | 12.81 | 10.80 | 8.75 |
| Tyrosine | 1.14.16.1 | phenylalanine-4-hydroxylase | ko00400 | 0.47 | 0.80 | 1.66 | 1.09 | 0.46 | 0.49 | 19.36 | 8.31 | 5.94 | 1.73 | 4.11 | 1.73 |
| Leucine,isoleucine,valine | 2.2.1.6 | acetolactate synthase | ko00290 | 377.66 | 484.44 | 437.32 | 323.37 | 230.32 | 188.81 | 665.31 | 644.32 | 492.37 | 478.06 | 529.15 | 382.06 |
|  | 1.1.1.86 | ketol-acid reductoisomerase | ko00290 | 106.11 | 68.61 | 68.22 | 70.64 | 50.77 | 44.92 | 218.04 | 228.70 | 162.05 | 166.36 | 196.16 | 139.17 |
|  | 4.2.1.9 | dihydroxy-acid dehydratase | ko00290 | 243.71 | 97.65 | 160.75 | 131.35 | 81.72 | 70.00 | 370.16 | 435.80 | 328.95 | 319.00 | 365.89 | 234.01 |
|  | 2.6.1.42 | branched-chain amino acid aminotransferase | ko00290 | 240.44 | 172.52 | 195.84 | 322.62 | 342.57 | 331.70 | 278.64 | 329.06 | 310.67 | 330.17 | 357.77 | 296.43 |
|  | 4.2.1.33 | 3-isopropylmalate dehydratase | ko00290 | 187.23 | 123.94 | 132.75 | 164.01 | 113.51 | 89.09 | 446.70 | 445.83 | 335.92 | 350.93 | 411.57 | 250.20 |
|  | 1.1.1.85 | 3-isopropylmalate dehydrogenase | ko00290 | 194.08 | 68.06 | 103.22 | 80.66 | 55.69 | 48.37 | 226.29 | 237.03 | 175.79 | 173.02 | 204.11 | 127.14 |
